# Seasonal and interpopulational phenotypic variation in morphology and sexual signals of *Podarcis liolepis* lizards

**DOI:** 10.1371/journal.pone.0211686

**Published:** 2019-03-15

**Authors:** Jesús Ortega, José Martín, Pierre-André Crochet, Pilar López, Jean Clobert

**Affiliations:** 1 Department of Evolutionary Ecology, National Museum of Natural Sciences, C.S.I.C., Madrid, Spain; 2 Department of Biology, Lund University, Lund, Sweden; 3 Centre d’Ecologie Fonctionnelle et Evolutive, Montpellier, CNRS-UMR 5175, France; 4 Station d’Ecologie Théorique et Expérimentale, Moulis, France; University of Cambridge, UNITED KINGDOM

## Abstract

Widespread species often show extensive phenotypic variation due to the contrasting abiotic and biotic factors that shape selective pressures in different environments. In this context, the gradual and predictable patterns of variation in climatic and environmental conditions found in mountain areas offer a great opportunity to explore intraspecific phenotypic variation. For instance, temperature is negatively correlated with altitude and virtually all aspects of the behavior and physiology of ectotherms are sensitive to body temperature. In this work, we tested the hypothesis that morphology, dorsal and ventral coloration and the chemical profile of femoral secretions show interpopulational and seasonal variation in the lacertid lizard (*Podarcis liolepis*). We compared lizards from three populations inhabiting lowland and highland habitats in the French Pyrenees that were closely related genetically. We found that highland lizards were larger, stockier, had longer heads and more femoral pores and had a darker dorsal coloration than lowland ones. In addition, we detected interpopulational differences in both the abundance and the richness of chemical compounds in the glandular secretions, and we also found seasonal variation in the overall chemical composition. Dorsal and ventral coloration differed seasonally and between populations. Ventral and dorsal brightness were higher in lowland than in highland lizards in the reproductive season whereas the reversed trend was found in the non-reproductive season but only for dorsal brightness. In addition, all lizards had browner dorsal coloration in the non-reproductive season, and lowland lizards were greener in the reproductive season. By integrating information from both visual and chemical systems, our works offers a comprehensive view of how these lizards communicate in a multimodal context.

## Introduction

Understanding patterns of geographical variation within species is of interest since the mechanisms may be similar to those responsible for divergence between species [1–3]. Abiotic variables can drive spatial and/or temporal variation in organismal traits [4–5] and populations of the same species inhabiting distinct localities can experience contrasting ecological and climatic conditions that, ultimately give rise to phenotypic divergence [6–7]. Hence, studying widespread species that occupy different environments and show extensive phenotypic variation has improved our understanding of the link between environmental and phenotypic variation [8–10]. Specifically, elevational differences found in mountain areas bring about gradual and predictable patterns of variation in climatic and environmental conditions on a small geographical scale, e.g. temperature decreases while humidity increases with elevation. In this context, the ecology of ectotherms, such as lizards, should be particularly affected by elevation because so much of their biology is temperature dependent [11–12]. Thus, lizards distributed over a wide elevational range may exhibit phenotypes, such as body size [13], body coloration [14], and visual [15] and chemical signal design [16], that are either the result of phenotypic plasticity and/or local adaptation, generating clines for several traits with elevation.

Bergmann’s rule states that larger animal phenotypes are associated with colder climates [17–20]. The underlying mechanism for this pattern is the 'heat conservation hypothesis', in which larger individuals need to produce less heat to maintain core body temperature over environmental temperature [21]. Hence, it has been suggested that Bergmann’s rule applies primarily to endothermic species [22] and several studies on frogs, lizards and snakes suggested that a smaller body size in colder climates might enable a more efficient thermoregulation, with fast warming being especially beneficial at higher elevations [23, 24, 25]. However, research in lizards has yielded puzzling results as many species exhibit reversed Bergmann´s clines [23] while others follow it [13] and different trends can be found within a genus [21, 26] or even within the same species [27–28].

Besides the impact of altitude on body size, ectotherms tend to be darker at higher elevations, and several hypotheses are proposed to explain this observation. The ‘thermal melanism hypothesis’ postulates that darker individuals have thermoregulatory advantages (fast heating rates) and, hence, they should be favoured in habitats with low temperatures [29–30]. A second hypothesis that could explain why organisms darken with altitude is based on the increasing UV radiation intensity with elevation (‘protection against UV damage hypothesis’, [14, 31]). Alternatively, the ‘camouflage hypothesis’ states that predation may select for cryptic coloration that renders individuals inconspicuous against their visual backgrounds [32–34]. Thus, as coloration conspicuousness is a combined function of ambient light and background reflectance and both variables are influenced by altitude [35], dorsal coloration should vary with elevation as a way to reduce predation by background matching.

Signals may also show intraspecific variation: their evolution results from the balance between natural and sexual selection [36–37] and are also expected to show phenotypic plasticity to maximize efficacy in a given environment [38–39]. Environmental conditions, which covary with geography and microhabitat, may influence signal detectability, durability or persistence and constitute selective factors for the efficacy of sexual signals [40–43]. The divergence of mating signals and preferences likely occur because geographically distinct populations experience different environmental conditions that can influence the strength or direction of sexual selection [6]. Consequently, signals are expected to vary among populations to remain informative despite geographical variation in environmental conditions. As the environmental conditions that influence signal detectability, durability or persistence may also change on a temporal basis, we also expected seasonal variation between reproductive and non reproductive seasons in the properties of the signals to cope with contrasting levels of, for example, light, humidity or temperature.

Chemoreception is one of the main sensory systems in lizards, and chemical signals play an important role in communication and sexual selection in these animals [44–47]. For example, chemical signals from femoral glands or faeces are very often used to scent-mark substrates allowing the delimitation of territories or attract mates [48–49] and they may play a key role in lizard recognition, hierarchy establishment, or mate choice [49–52]. In the context of communication optimization, chemical signals used for scent marking should ensure efficiency by appropriately tuning to environmental factors such as local humidity and temperature that affect their volatility and, therefore, their persistence and transmission through the environment [53–54]. However, their seasonal [55] and intraspecific variability have been rarely explored in lizards [56–61]. Besides chemical signals, many lizards have also evolved colourful sexually dimorphic ornaments that are used in intraspecific relationships [62]. The condition dependence and sexually selected nature of carotenoid- and pteridine-based ventral coloration suggest that these ornaments would be particularly variable among populations [63–64] and can be tailored to the ambient light and microhabitat conditions that vary with elevation and season.

In the present work, we specifically examined a range of phenotypic traits: morphology, dorsal and ventral coloration, and femoral secretion composition of two *P*. *liolepis* populations in the French Pyrenees during the reproductive and the non-reproductive seasons. For morphological comparisons, we included a third population from the same region that could not be surveyed in both seasons. We also assessed the genetic relatedness of the populations to verify that we are not sampling different genetic lineages that could confound the phenotypic differences observed. We tested the hypothesis that morphology, number of femoral pores, dorsal and ventral coloration, and the chemical profile of femoral secretions show intraspecific variation in *P*. *liolepis*. Some lacertid lizard species are larger [25, 65] and have more femoral pores at higher altitudes [57]. In a similar fashion, it has been shown that lizards are darker as the altitude increases [13]. Thus, we first predicted that *P*. *liolepis* inhabiting the high elevation site would be larger, darker and would have more femoral pores than lizards at low elevation. A similar pattern has also been found in *P*. *guadarramae*, which is part of this species complex [57, 65, 66]. In the context of communication optimization and given the contrasted climatic conditions and physical properties of highland and lowland environments, we also predicted interpopulational and seasonal differences in chemical and visual sexual signals (i.e. femoral secretions and ventral coloration). We expected contrasted chemical profiles in femoral secretions between populations and between the reproductive and the non-reproductive seasons [55, 61]. We also predicted more conspicuous ventral coloration during the reproductive season. Finally, we expected contrasted dorsal coloration between populations and seasons in keeping with the ‘camouflage hypothesis’.

## Material and methods

### Study species

The Brown lizard *Podarcis liolepis* is a small lacertid recently recognised as a species within the *Podarcis hispanicus* species complex [67, 68]. It is distributed along the Mediterranean Spanish coast from Valencia to Catalonia, westwards to the Basque Country, the Ebro Valley and the Castilian plateau; and in southern France east to the Rhone river [67– 69]. *Podarcis 'hispanicus'* is a widespread species complex of lacertid lizards in which genetic and phenotypic variation are extremely high both within and among lineages or species [70–73].

During 4^th^-11^th^ of May and 28^th^ September-12^th^ October 2012 (hereafter reproductive and non-reproductive season, respectively) we captured by noosing sexually mature male and female lizards at several populations in Ariège, in the region of the Midi-Pyrenees, France. `Pêch de Foix´ (42°57'46.79"N, 1°37'22.17"E; 800 m altitude) and `Vaychis´ (42°44'53.20"N, 1°50'25.58"E; 1400 m altitude) populations. A third population in `Le Mas d´Azil´ (43° 4'22.17"N, 1°21'29.47"E; 400 m altitude) could only be surveyed in the non-reproductive season for logistic reasons. Hereafter, we will use the names Foix, Azil (lowland sites) and Vaychis (highland site) for brevity.

All animals were healthy and were returned to their capture sites at the end of trials. Captures of lizards and field studies were performed under license (permit number: SA-013-PB-092) from the Direction Départementale de La Cohésion Sociale et de La Protection des Populations, Service Sante protection Des Animaux et Environnement (Ariège region). The Animal Ethics Committe of the Station d’Ecologie Théorique et Expérimentale evaluated this research and granted a waiver of ethics approval given the absence of experimental manipulation.

### Climatic data

We extracted mean temperatures and total precipitation per month during 1998–2012 from the Global Climate Monitor (http://www.globalclimatemonitor.org). The data available comes from multiple sources: the CRU database produced by the Climate Research Unit (University of East Anglia), the Global Precipitation Climatology Centre (GPCC) and the Global Historical Climatology Network-Monthly (GHCN-M). Differences in mean temperatures and total precipitation between populations were investigated using General Linear Models (GLMs) with population and month as fixed factors.

### Morphological measurements

We measured body size of lizards using a ruler (snout-vent length, ‘SVL’; to the nearest 1 mm). We used a digital caliper to measure (to the nearest 0.05 mm) the following morphological variables: ‘head length’ was the distance between the tip of the snout and the posterior side of the parietal scales; ‘head width’ was the greatest distance between the external sides of the parietal scales; ‘head height’ was the greatest distance from the highest portion of the head to the bottom of the lower jaw. ‘Femoral length’ was the mean distance from the hip to the knee measured in both hindlimbs. A magnifying glass was used to count the number of femoral pores on the ventral side of left and right thighs.

When initial GLM models showed non-significant interactions, these were excluded from further morphological analyses (all *P* values > 0.099).

### Coloration measurements

We used an Ocean Optics USB 2000 spectrophotometer to measure the reflectance of lizard coloration using a dual DT-1000-MINI Deuterium–Halogen light source (Ocean Optics, Inc., Dunedin, FL, USA). All lizards were kept in the Station d’Ecologie Théorique et Expérimentale for at least one hour to avoid the effect of temperature differences among individuals in coloration measurements. A custom-made probe holder oriented at 45 degrees and 1 cm away from the skin surface was used to exclude ambient light and standardize measuring distance. Each spectral reading consisted of percent reflectance recordings in reference to a white standard. Spectral raw data were processed with CLR 1.1. software [74] and reflectance readings from 300 to 700 nm, summarized over 5 nm steps [75], were selected for analysis, as they represent the spectral sensitivity measured for other lizard species [74–79] and their avian predators [80].

Conspicuous sexual signals are located on body surfaces visible to conspecifics (e.g. ventrolateral region) and less visible to predators, while camouflage is found on regions more exposed to predators (e.g. dorsal region) [32–33]. Thus, we measured coloration of dorsal and ventral body areas. Dorsal coloration was measured at two mid-dorsal standardized sites: above the scapular hip (‘proximal dorsal’) and above the pelvic hip (‘caudal dorsal’). On the other hand, ventral coloration was measured at four standardized sites: throat (between the last chin shields and the collar; ‘throat’), breast (just anterior to the two forelimbs at the middle of the second row of scales prior to the collar; ‘breast’), in the abdominal area (‘belly’; in the middle point between ‘breast’ and ‘precloacal’), and the precloacal area (in the middle point before the two forelimbs; ‘precloacal’). As initial analyses did not show significant differences between the two measures taken in the dorsal region or between the four of the ventral region (all *P´s* > 0.963), we averaged spectral data for each body region.

**Fig 1:**
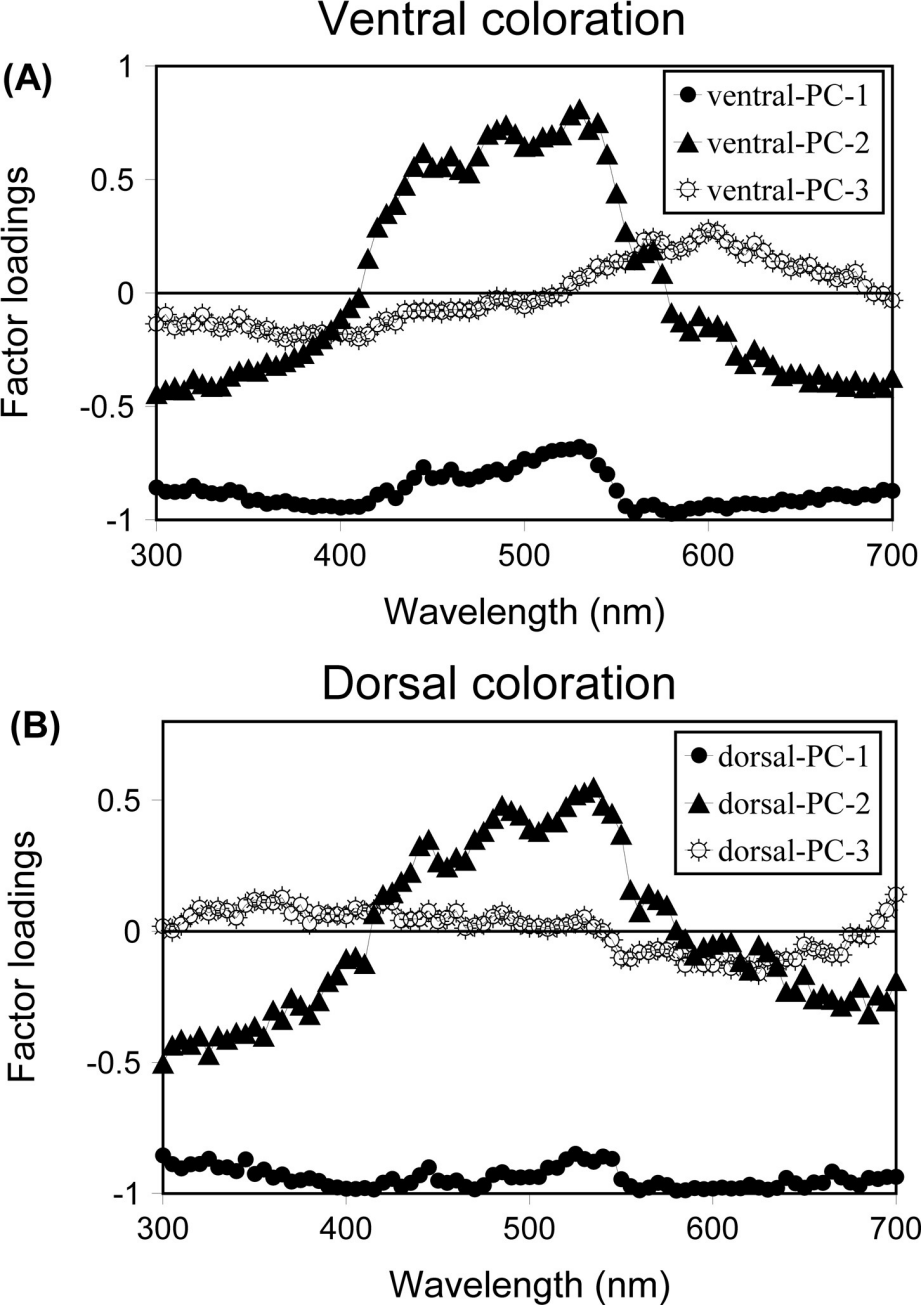
Coefficients of the first three principal components from PCAs on reflectance spectra that characterize (A) ventral and (B) dorsal coloration of two *P*. *liolepis* populations in the French Pyrenees.

Principal components analyses were performed with coloration measurements for males and females in both seasons but separately for dorsal and ventral areas. The PCA summarizes all of the information about the shape of complex reflectance spectra, including bimodal ones, such as those found here. In PCA of spectral data, PC1 represents variation in the intensity of coloration or brightness, and subsequent PCs represent combinations of hue and chroma [75, 79–81] (Fig 1). Also, the PCA identifies those sections of the spectrum (wavelength regions) that are contributing to the observed variation, independently of their importance in terms of contribution to the total amount of reflectance [74].

We used General Lineal Models (GLMs) with each of the PC scores describing coloration as dependent variables and with ‘population’ and ‘season’ (the reproductive season vs. the non-reproductive season) as fixed factors and including the interactions between effects in the models. Body size (SVL) was included as a covariate in the models as body coloration changes with ontogeny and, hence with body size [82].

### Chemical signals of lizards

We extracted femoral gland secretion of male lizards from both populations by gently pressing around the femoral pores, and collected secretion directly in glass vials with glass inserts (reproductive season: Vaychis = 5, Foix = 10; non-reproductive season: Vaychis = 8, Foix = 9). We also used the same procedure but without collecting secretion, to obtain blank control vials that were treated in the same manner to compare with the actual samples. Samples were stored at -20°C until analyses. Prior to the analytical process, we examined the repeatability of the GC-MS process with five consecutive injections of a standard compound (heptadecane) made on different days, being the relative standard deviation (RSD) always below 1 for retention time and 10 for peak areas.

Samples were analyzed using a Finnigan-ThermoQuest Trace 2000 gas chromatograph (GC) fitted with a poly (5% diphenyl/ 95% dimethylsiloxane) column (Supelco, Equity-5, 30mlength x 0.25mmID, 0.25-μmfilm thickness) and a Finnigan-ThermoQuest Trace mass spectrometer (MS) as a detector. Sample injections (2 μl of each sample dissolved in 200 μl of n-hexane; Sigma, capillary GC grade) were performed in splitless mode using helium as the carrier gas, with injector and detector temperatures at 270°C and 250°C, respectively. The n-hexane (99%) for organic residue analysis was obtained from J.T. Baker (Deventer, The Netherlands). The oven temperature program was as follows: 50°C isothermal for 10 min, then increased to 280°C at a rate of 5°C/min, and then isothermal (280°C) for 20 min. Mass spectral fragments below m/z = 39 were not recorded. Impurities identified in the solvent and/or the control vial samples were not reported. Data recording began 7 min after the separation initiated using the Software XcaliburTM 1.4 (Thermo Fischer Scientific Inc., San Jose, CA, USA). Impurities identified in the control vial samples were not considered.

Initial identification of secretion components was done by comparison of mass spectra in the NIST/EPA/NIH 2002 computerized mass spectral library. When possible, identifications were confirmed by comparison of spectra and retention times with those of authentic standards. Authentic samples were purchased from Sigma-Aldrich Chemical Co. We identified and calculated relative proportions determined as the percent of the total ion current (TIC) of major compounds (>1% of the TIC area) in secretions. We compared the relative proportion of compounds found in femoral secretions between populations and seasons. To correct the problem of non-independence of proportions, we transformed areas using the compositional data analysis, where the transformed proportion = Log((original proportion / (1 –original proportion)) [83]. Then, we calculated Euclidean distances between every pair of individual samples to produce a resemblance matrix that formed the basis of the analyses. To compare the entire chemical profiles, we used a two-way permutational multivariate analysis of variance test (PERMANOVA) [84–85] based on the Euclidean resemblance matrix using 999 permutations. All the identified compounds were included in the analyses. Differences were investigated further using a canonical analysis of principal coordinates (CAP) [86]. GLMs were used to test for differences in proportions of specific compounds and categories. The software PRIMER V6.1.13 [87] with the PERMANOVA+ V1.0.3 add-on package [88] was used to investigate differences between chemical profiles. GLMs and Tukey´s honestly significant difference tests in post hoc pairwise comparisons [89] were performed in this and former sections with the STATISTICA v8 software.

### Genetic analyses

DNA from 34 *P*. *liolepis* lizards from the studied populations was extracted by complete digestion of a piece of tail muscle using the DNeasy Blood and Tissue kit following the manufacturer’s recommended procedures (Spin-Column Protocol, Qiagen). In order to assign individuals to a mitochondrial DNA lineage, we sequenced the ND4 gene in all analyzed individuals. Polymerase chain reaction (PCR) amplifications were performed in a 10 μl reaction volume containing 1 μl DNA solution, 5 μl of Quiagen Multiplex Kit and 1 μl of primers at 2 μM. PCRs started with an initial denaturation step at 95°C for 5 min, followed by 40 cycles of denaturation at 94°C for 1 min, annealing at 58°C for 90 s and extension at 72°C for 1 min. All reactions were finished with a final extension at 72°C for 7 min. Successful PCRs were selected on ethidium-bromide stained agarose gels.

All sequences were obtained for both the reverse and forward primers. Alignment was performed manually using CodonCode Aligner 3.9 (Codon Code Corporation) and automatic base calls were checked along the sequence, both where the two sequences were in disagreement and elsewhere. Genetic structure based on the ND4 mitochondrial marker was estimated with Maximum-likelihood based on the Tamura-Nei model [90] in MEGA, version 4.1 [91].

## Results

### Climatic conditions

The mean air temperatures increased significantly within a given year from January to August in both populations and reached the greatest values in July and August (GLM; month: *F*_11,504_ = 1023.58, *P* < 0.001; locality: *F*_2,504_ = 900.63, *P* < 0.001; month x locality: *F*_22,504_ = 0.47, *P* = 0.981), and Vaychis had lower temperatures than Foix and Azil (Tukey´s post hoc comparisons; *P´s* < 0.001) and the difference between the latter populations was not significant (*P* = 0.275). Total precipitation was highly variable between months, decreasing significantly from May to July, and increasing from July to October (GLM; month: *F*_11,504_ = 8.66, *P* < 0.001; locality: *F*_2,504_ = 9.43, *P* < 0.001; month x locality: *F*_22,504_ = 0.37, *P* = 0.996). Total precipitation was lower in Azil than in Vaychis and Foix (all *P* values > 0.032) but the difference between Vaychis and Foix was not significant (*P* = 0.165). We found temperature but not precipitation differences between the reproductive and the non-reproductive season. Mean temperature in May was higher than in October (*P* = 0.009) but total precipitation, although higher in May, was not significantly different between May and October (*P* = 0.971).

### Morphology

Overall, highland lizards were bigger, had more robust heads (wider and taller heads for the same length) and longer femora than those from the two lowland populations (GLM for SVL: sex: *F*_1,65_ = 4.13, *P* = 0.038; population: *F*_2,65_ = 0.13, *P* = 0.002). The same result was found for males in comparison to females in all populations (Tables 1 and 2). The only variable that did not significantly differ among populations was head length, but males had longer heads than females. The significant interaction showed that females from Vaychis had shorter heads than males but did not significantly differ from females from other populations (GLM for head length: sex: *F*_1,63_ = 21.85, *P* < 0.001; population: *F*_2,63_ = 1.46 *P* = 0.241; sex x population: *F*_2,63_ = 4.04, *P* = 0.022) (Tables 1 and 2). Highland lizards and males had more femoral pores than lowland and female lizards, respectively (Tables 1 and 2) and these differences remained significant even after controlling for femoral length differences among populations (GLM with femoral length as a covariate; femoral length: *F*_1,62_ = 0.15, *P* = 0.700; sex: *F*_1,64_ = 7.58, *P* = 0.008; population: *F*_2,64_ = 5.03, *P* = 0.009). When initial GLM models showed non-significant interactions, these were excluded from further morphological analyses (all *P* values > 0.099).

**Table 1:** Mean (±1 SE) values for morphological variables of adult *P*. *liolepis* lizards from highland and lowland populations in the French Pyrenees. The means that share the same small letters are not significantly different among them (post-hoc Tukey's tests, *P* < 0.05), but they are significantly different of means with a different letter."

Altitude (m)	Pêch de Foix 800		Vaychis 1400		Le Mas d´Azil 400	
	Females	Males	Females	Males	Females	Males
	*n* = 15	*n* = 22	*n* = 5	*n* = 13	*n* = 6	*n* = 8
SVL (mm)	50.2 ± 1.2^a^	52.4 ± 1.0^a^	55.4 ± 2.1^b^	58.0 ± 1.3^b^	50.3 ± 1.9^a^	54.3 ± 1.7^a^
Head length (mm)	9.4 ± 0.3^a,b^	11.3 ± 0.3^a^	8.3 ± 0.6^b^	12.3 ± 0.4^a^	9.1 ± 0.5^a,b^	11.1 ± 0.5^a^
Head width (mm)	5.8 ± 0.2^a^	7.1 ± 0.1^a^	6.3 ± 0.3^b^	7.8 ± 0.2^b^	5.6 ± 0.2^a^	6.9 ± 0.2^a^
Head height (mm)	4.1 ± 0.1^a^	4.7 ± 0.1^a^	4.3 ± 0.2^b^	5.3 ± 0.1^b^	3.8 ± 0.2^a^	4.6 ± 0.1^a^
Femoral length (mm)	7.3 ± 0.2^a^	8.7 ± 0.2^a^	8.2 ± 0.4^b^	10.1 ± 0.2^b^	7.1 ± 0.3^a^	8.7 ± 0.3^a^
Femoral pore number	16.2 ± 0.2^a^	17.1 ± 0.2^a^	16.7 ± 0.4^b^	18.2 ± 0.3^b^	16.1 ± 0.4^a^	16.6 ± 0.3^a^

**Table 2:** Effect of population, sex and their interaction on morphology in three *P*. *liolepis* populations in the French Pyrenees. SVL was added as a covariate to statistically remove the effect of body size. Significant probabilities are marked in bold.

		Head length		Head width		Head height		Femoral length		Femoral pore number	
	*df*	*F*	*P*	*F*	*P*	*F*	*P*	*F*	*P*	*F*	*P*
SVL	1, 62	3.45	0.068	151.08	**< 0.001**	60.02	**< 0.001**	144.38	**< 0.001**	0.13	0.723
Sex	1, 62	17.26	**< 0.001**	160.80	**< 0.001**	62.79	**< 0.001**	98.69	**< 0.001**	12.17	**< 0.001**
Population	2, 62	2.73	0.073	7.61	**0.001**	7.47	**0.001**	7.17	**0.002**	6.27	**0.003**
Sex x population	2, 62	4.31	**0.018**	-	-	-	-	-	-	-	-

### Ventral coloration

The PCA on reflectance data of all spectra of ventral coloration produced three principal components (PCs) that together accounted for 96.52% of the variation in the original spectra (Fig 1A and S1 and S2 Tables). The first PC (ventral-PC1) accounted for 74.72% of variation (eigenvalue = 60.53). Coefficients relating ventral-PC1 to the original reflectance data were all negative and of similar magnitude, so ventral-PC1 represented achromatic brightness variation in the original spectra. The second PC (ventral-PC2) accounted for a further 19.77% of the variation (eigenvalue = 16.01) in the original spectra and the pattern of coefficients suggested that positive values represented variation in both short and medium (415–575 nm) wavelengths while negative values reflect variation in both very short (300–410 nm) and very long (580–700 nm) wavelengths. Thus ventral-PC2 represented variation in the relative amount of very short and very long wavelengths to medium wavelength reflectance, with greater PC2 scores indicating more saturated ‘greenish’ colours. The third PC (ventral-PC3) accounted for 2.03% of the variation (eigenvalue = 1.64). The coefficients relating the ventral-PC3 to the original reflectance values below 520 nm were all negative, while above 520 nm they were positive, except for 690–700 nm which were negative. Thus, more positive ventral-PC3 scores indicated more saturated ‘reddish’ colours.

**Fig 2:**
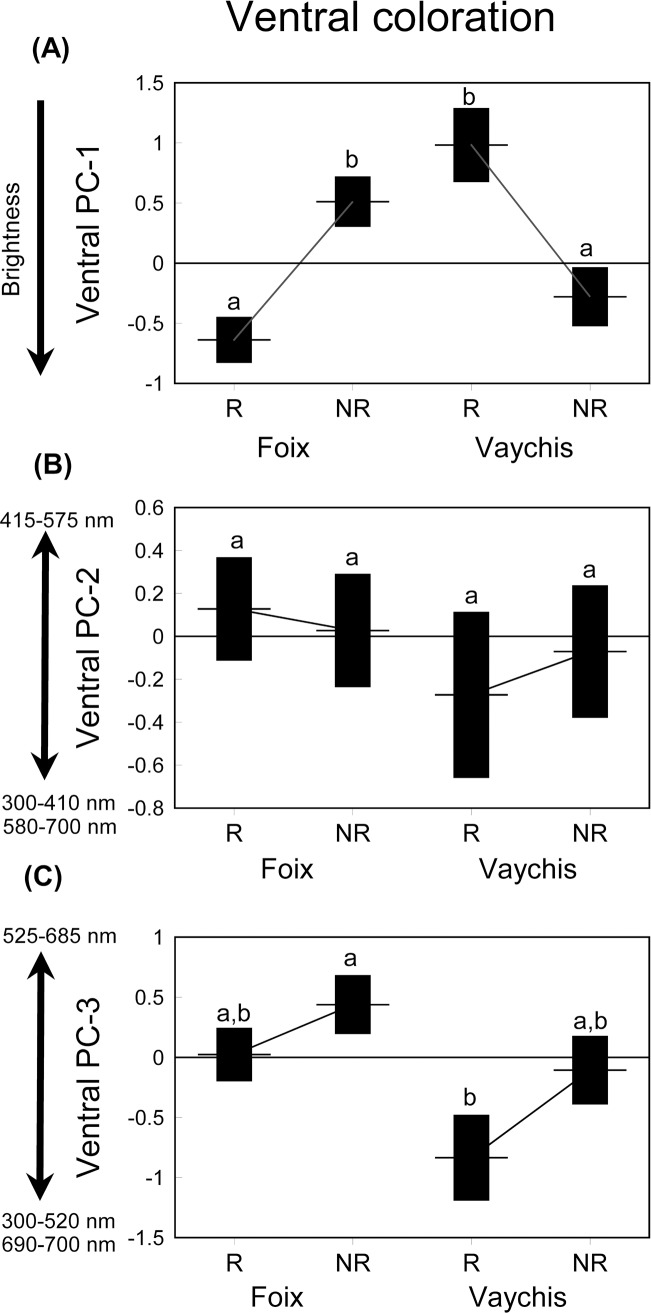
Mean ± SE values of ventral-PC scores describing ventral coloration. (A) Brightness (PC1), (B) greenish (PC2) and (C) reddish coloration (PC3) of *P*. *liolepis* sampled in the reproductive (R: May) and non-reproductive (NR: October) seasons. The means that share the same small letters are not significantly different among them (post-hoc Tukey's tests, *P* < 0.05), but they are significantly different of means with a different letter.

Initial GLM models showed that the effects of the interactions involving fixed factors described by the three ventral PCs were not significant (all *P* values > 0.099), except in the PC1 for the population x season interaction. Thus, we excluded the non-significant interactions from further analyses. Overall ventral brightness (PC1) did not differ between populations, seasons or sexes. However, lizards were brighter in the lowland than in the highland population in the reproductive season whereas there were not significant differences in the non-reproductive season (Table 3 and Fig 2A). This result remained significant after controlling for body size (GLM with SVL as a covariate; SVL: *F*_1,45_ = 1.81, *P* = 0.184; population: *F*_1,45_ = 0.69, *P* = 0.409; season: *F*_1,45_ = 0.37, *P* = 0.548; sex: *F*_1,45_ = 0.01, *P* = 0.952; population x season: *F*_1,45_ = 0.69, *P* <0.001). Greenish coloration (PC 2) did not show any significant overall difference between, populations, seasons or sexes (Table 3 and Fig 2B). Nonetheless, lizards in the lowland population and the non-reproductive season were redder than those from the highland population and the reproductive season, respectively (PC3) (Table 3 and Fig 2C), however, only the differences between populations remained significant after controlling for body size (GLM with SVL as a covariate; SVL: *F*_1,46_ = 1.15, *P* = 0.289; population: *F*_1,46_ = 7.70, *P* = 0.008; season: *F*_1,46_ = 2.60, *P* = 0.114; sex: *F*_1,46_ = 0.63, *P* = 0.430).

**Table 3:** Effect of population, season, sex and their interactions on ventral and dorsal coloration in two *P*. *liolepis* populations in the French Pyrenees. Significant probabilities are marked in bold.

	Ventral						Dorsal					
	PC1		PC2		PC3		PC1		PC2		PC3	
	*F*_1,46_	*P*	*F*_1,47_	*P*	*F*_1,47_	*P*	*F*_1,46_	*P*	*F*_1,47_	*P*	*F*_1,47_	*P*
Population	2.69	0.107	0.48	0.493	6.60	**0.013**	3.29	0.077	9.39	**0.004**	2.65	0.110
Season	0.04	0.842	0.01	0.972	4.19	**0.046**	2.95	0.093	9.49	**0.003**	15.23	**< 0.001**
Sex	0.05	0.821	0.28	0.599	1.09	0.303	0.38	0.541	0.45	0.506	0.06	0.809
Population x season	23.66	**< 0.001**	-	-	-	-	44.71	**< 0.001**	-	-	-	-

### Dorsal coloration

The PCA on reflectance data of all spectra of dorsal coloration produced three principal components (PCs) that together accounted for 96.35% of the variation in the original spectra (Fig 1B and S3 and S4 Tables). The first PC (PC1) accounted for 86.44% of variation (eigenvalue = 70.02). Coefficients relating dorsal-PC1 to the original reflectance data were all negative and of similar magnitude, so dorsal-PC1 represented achromatic brightness variation in the original spectra. The second PC (dorsal-PC2) accounted for a further 11.10% of the variation (eigenvalue = 9.00) in the original spectra and the pattern of coefficients suggested that positive values represented variation in both short and medium (415–610 nm) wavelengths while negative values reflect variation in both very short (300–410 nm) and very long (615–700 nm) wavelengths. Thus dorsal-PC2 represented variation in the relative amount of short, UV to long, visible, wavelength reflectance, with greater PC2 scores indicating more ‘greenish’ coloration. The third PC (dorsal-PC3) accounted for 0.81% of the variation (eigenvalue = 0.65). Regarding the coefficients relating dorsal-PC3 to the original reflectance, below 540 nm were all positive (see Fig 1B), while above 540 nm they were negative, except for 690–700 which were positive. Thus, more negative PC3 scores indicated more saturated ‘brownish’ dorsal coloration.

**Fig 3:**
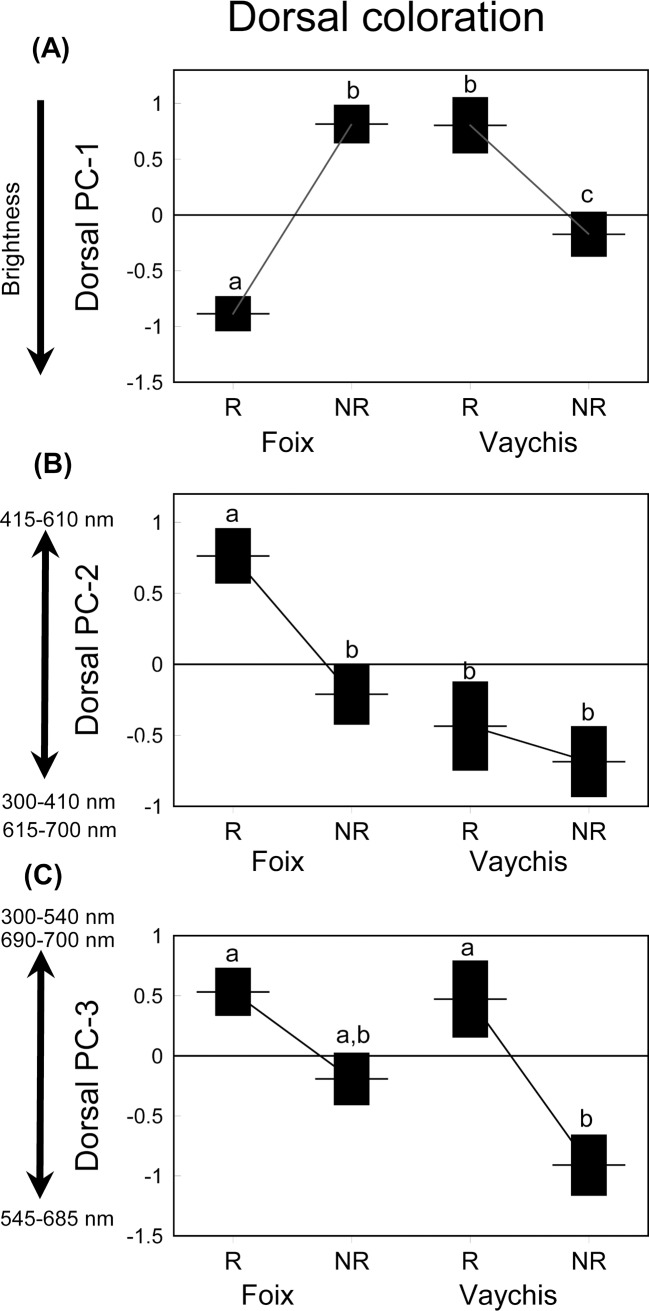
Mean ± SE values of dorsal-PC scores describing dorsal coloration. (A) Brightness (PC1), (B) greenish (PC2) and (C) brownish coloration (PC3) of *P*. *liolepis* sampled in the reproductive (R: May) and non-reproductive (NR: October) seasons. The means that share the same small letters are not significantly different among them (post-hoc Tukey's tests, *P* < 0.05), but they are significantly different of means with a different letter.

**Fig 4:**
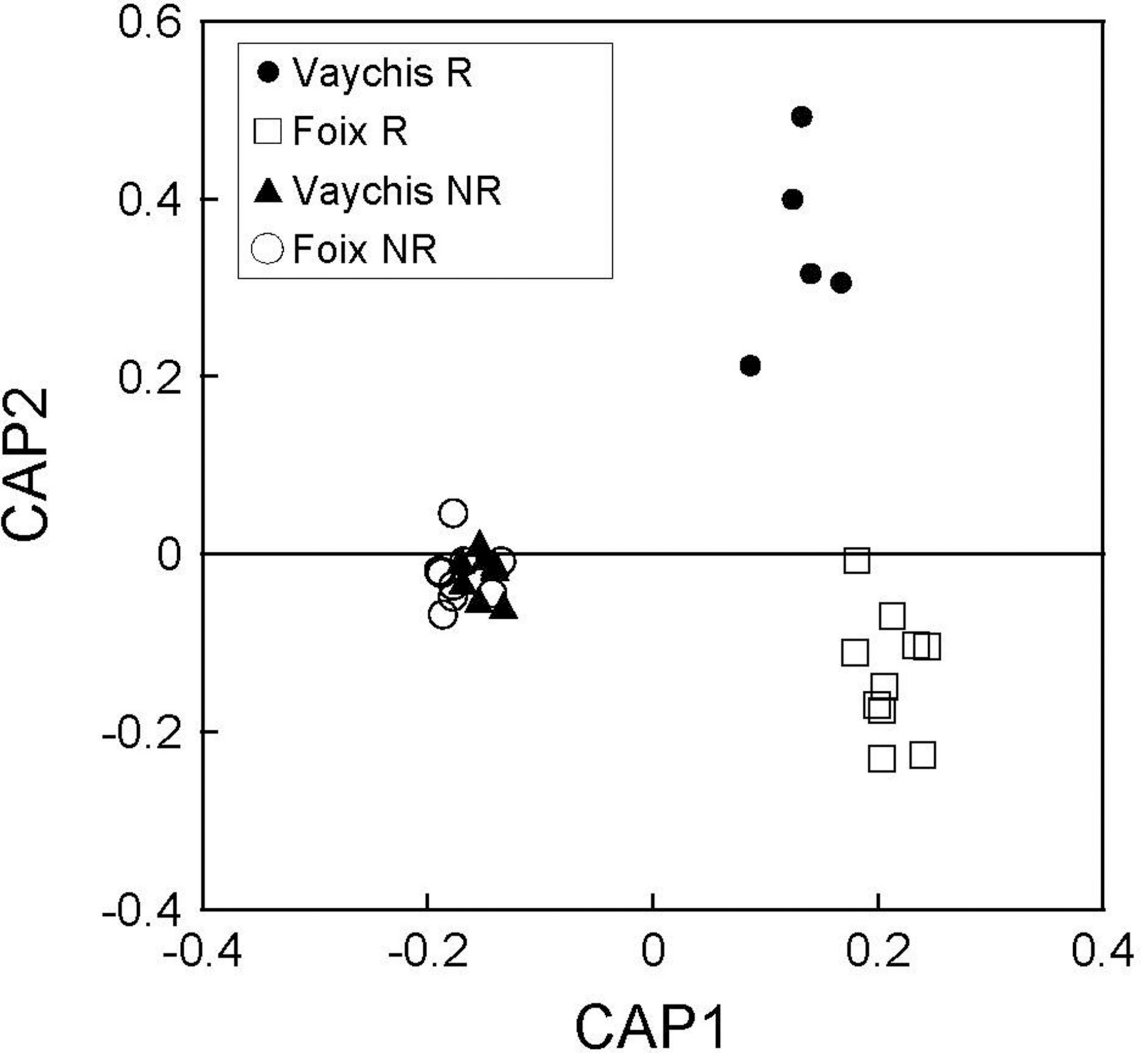
Representation of the first two axes of the canonical analysis of principal coordinates (CAP) showing classification of the femoral secretions of male *P*. *liolepis* of two populations in the French Pyrenees sampled in two seasons.

Initial GLM models showed that the effects of the interactions involving fixed factors described by the three PCs were not significant, except in the PC1 for the population x season interaction (all *P* values > 0.305). Thus, we excluded the non-significant interactions from further analyses. Dorsal brightness (PC1) did not significantly differ between populations, seasons or sexes but the population x sex interaction was significant (Table 3 and Fig 3A). According to these, lizards were brighter in the lowland than in the highland population during the reproductive season whereas the reverse trend was found in the non-reproductive season. This result remained significant after controlling for body size (GLM with SVL as a covariate; SVL: *F*_1,45_ = 1.26, *P* = 0.268; population: *F*_1,45_ = 1.18, *P* = 0.284; season: *F*_1,45_ = 1.68, *P* = 0.202; sex: *F*_1,45_ = 0.71, *P* = 0.405; population x season: *F*_1,45_ = 43.35, *P* <0.001). PC2 showed significant differences between populations and seasons (Table 3 and Fig 3B). Thus, lizards in the lowland population and the reproductive season had higher proportions of dorsal greenish coloration than in the highland population and the non-reproductive season, respectively, even after controlling for body size (GLM with SVL as a covariate; SVL: *F*_1,46_ = 1.14, *P* = 0.291; population: *F*_1,46_ = 10.30, *P* = 0.002; season: *F*_1,46_ = 10.65, *P* = 0.002; sex: *F*_1,46_ = 0.78 *P* = 0.383). PC3 showed significant differences between seasons, thus lizards showed more brownish dorsal coloration in the non-reproductive than in the reproductive season (Table 3), even after controlling for body size (GLM with SVL as a covariate; SVL: *F*_1,46_ = 0.28, *P* = 0.597; population: *F*_1,46_ = 1.37, *P* = 0.247; season: *F*_1,46_ = 12.33, *P* = 0.001; sex: *F*_1,46_ = 0.02 *P* = 0.902) (Table 3 and Fig 4C). S5 Table shows the summary statistics and sample sizes for both ventral and dorsal coloration analyses.

### Chemical signals

We found 142 lipophilic compounds in the femoral gland secretion of male *P*. *liolepis* (S6 Table). Overall, femoral secretions of males from the two populations were formed mainly by steroids (40.30% of the TIC areas, both populations combined), but there were also waxy esters (35.24%), terpenoids (15.91%), aldehydes (5.51%), fatty acids between C_4_ and C_20_ (1.91%), alcohols (0.91%), two ketones (0.14%) and tocopherol (0.03%). Considering specific compounds, squalene (14.93%) was the most abundant one, followed by cholesterol (12.15%).

There were clear differences in the presence/absence of some lipophilic compounds between *P*. *liolepis* populations. Both populations shared 71 out of the 142 detected compounds (50% of compounds, 93.50% of TIC) including all the main ones (i.e. those with proportions > 1%). *P*. *liolepis* from Vaychis had 48 exclusive compounds (33.9% of compounds; but only 4.05% of the TIC area) that were not found in *P*. *liolepis* from Foix which had 23 exclusive compounds (33.80% of compounds; but only 2.25% of the TIC area). However, reproductive and non-reproductive lizards only shared 32 compounds out of 142 (22.53% of compounds; 65.49% of the TIC area) but most of the shared compounds were the main compounds (S2 Table). During the reproductive season, lizards had 66 exclusive compounds (46.47% of compounds; 22.85% of the TIC area). In the non-reproductive season, lizards had 44 exclusive compounds (30.99% of compounds, 11.66% of the TIC).

We found significant differences in the chemical profiles (i.e. proportion of compounds) between populations and seasons (PERMANOVA; population: pseudo *F*_1,28_ = 11.48, *P* < 0.001; season: pseudo *F*_1,28_ = 31.02, *P* < 0.001; population x season: pseudo *F*_1,28_ = 10.01, *P* < 0.001) (post-hoc pairwise comparisons: all *P´s* < 0.004). The CAP analysis considering four groups (two populations separated into two seasons) (Fig 4) classified 96.87% of the individual chemical profiles into the correct population and season using leave-one-out cross-validation and m = 6 axes (δ_1_^2^ = 0.987, *P* = 0.001).

Comparing the different major categories of compounds, the lowland populations had significantly higher overall relative proportions of ketones, steroids and aldehydes whereas the highland populations had significantly higher relative proportions of fatty acids and their esters, waxy esters and terpenoids (Table 4). However, the proportion of alcohols was similar between both populations. There were not significant seasonal differences in the proportion of steroids, but in the reproductive season, lizards had significantly lower proportions of alcohols, fatty acids and their esters, ketones, aldehydes and terpenoids, and significantly higher proportion of waxy esters. The interaction terms showed that in the reproductive season, terpenoids, alcohols, ketones, steroids and aldehydes showed similar proportions between populations (Tukey´s post hoc comparisons; *P´s* > 0.622). However, in the non-reproductive season, proportions of terpenoids were significantly higher in the highland population (*P´s* < 0.001) and proportions of alcohols, ketones, steroids and aldehydes were significantly higher in the lowland population (*P´s* < 0.007 in all cases). However, seasonal differences between populations in the proportion of fatty acids and waxy esters were not significant (see Table 4).

**Table 4:** Effect of population, season, and their interactions on femoral secretion composition in two *P*. *liolepis* populations in the French Pyrenees. The different compound categories and the main specific compounds are shown. Significant probabilities are marked in bold.

	Population		Season		Population x season	
	*F*_*1*,*28*_	*P*	*F*_*1*,*28*_	*P*	*F*_*1*,*28*_	*P*
Compound categories						
Steroids	9.87	**0.004**	0.01	0.958	6.20	**0.019**
Waxy esters	5.04	**0.033**	49.62	**< 0.001**	3.72	0.064
Terpenoids	11.05	**0.002**	27.67	**< 0.001**	22.88	**< 0.001**
Aldehydes	18.79	**< 0.001**	98.91	**< 0.001**	18.79	**< 0.001**
Fatty acids and their esters	83.27	**< 0.001**	5.33	**0.029**	2.12	0.156
Alcohols	2.58	0.120	6.38	**0.018**	9.96	**0.004**
Ketones	7.94	**0.009**	7.94	**0.009**	21.51	**< 0.001**
Main specific compounds						
Cholesterol	4.80	**0.037**	12.18	**0.002**	1.16	0.290
Squalene	18.41	**< 0.001**	44.52	**< 0.001**	13.33	**0.001**

**Fig 5:**
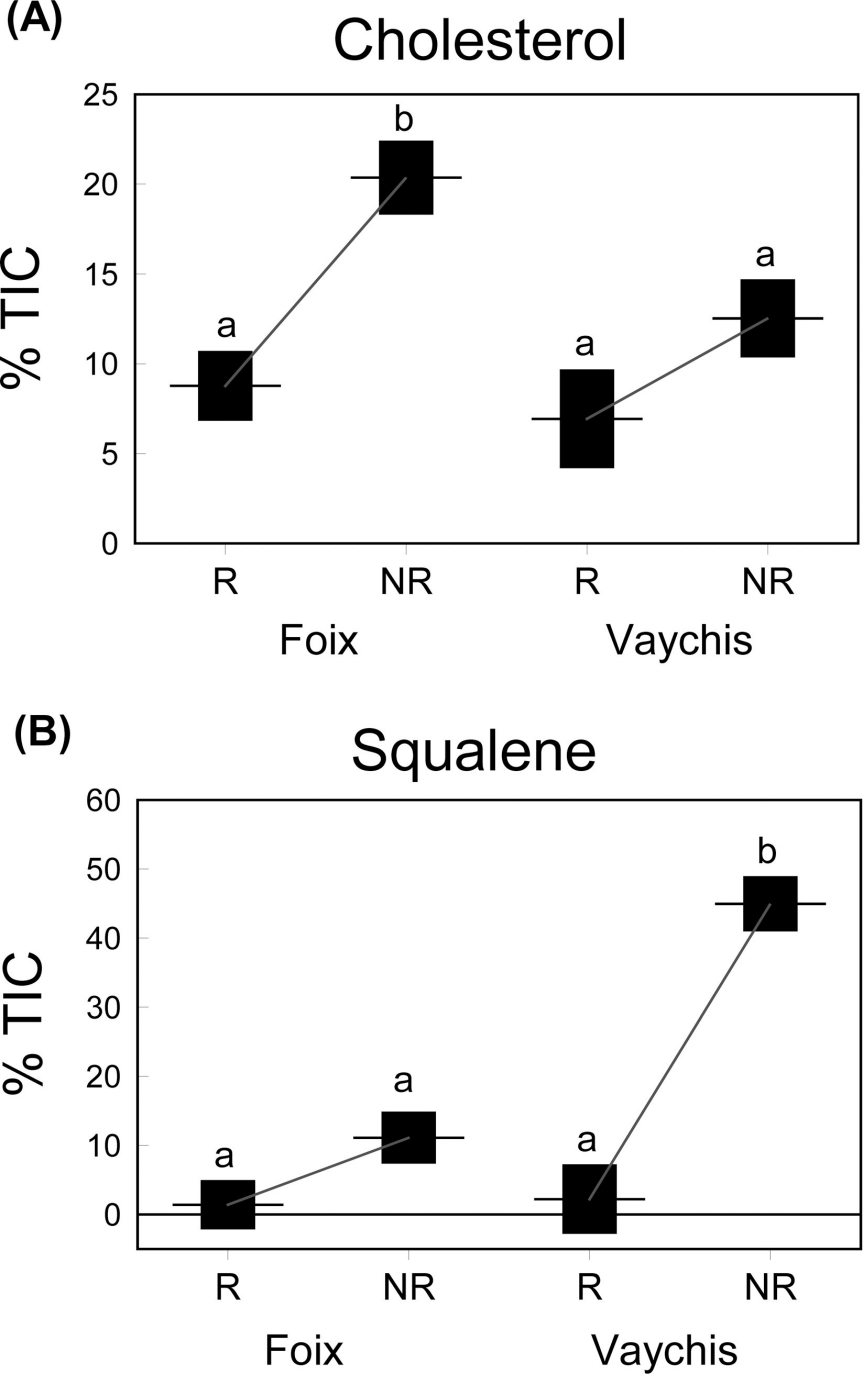
Interpopulational and seasonal differences in total ion current proportions (TIC) of (A) cholesterol and (B) squalene. TIC proportions (mean ± SE) of compounds from male femoral gland secretions of *P*. *liolepis* sampled in the reproductive (R: May) and non-reproductive (NR: October) seasons. The means that share the same small letters are not significantly different among them (post-hoc Tukey's tests, *P* < 0.05), but they are significantly different of means with a different letter."

Moreover, with respect to the two main compounds, relative proportions of cholesterol were significantly higher in the non-reproductive season and the lowland population; in particular, in the reproductive season, proportions of cholesterol were similar between populations, whereas in the non-reproductive season proportions of cholesterol were much higher in the lowland than in the highland population (Fig 5A and Table 4). We found that squalene was more abundant in the non-reproductive season and the highland population; specifically, in the reproductive season, the proportion of squalene was similar between populations, but, in the non-reproductive season, it was much higher in the highland than in the lowland population (Fig 5B and Table 4).

### Genetic analyses

**Fig 6:**
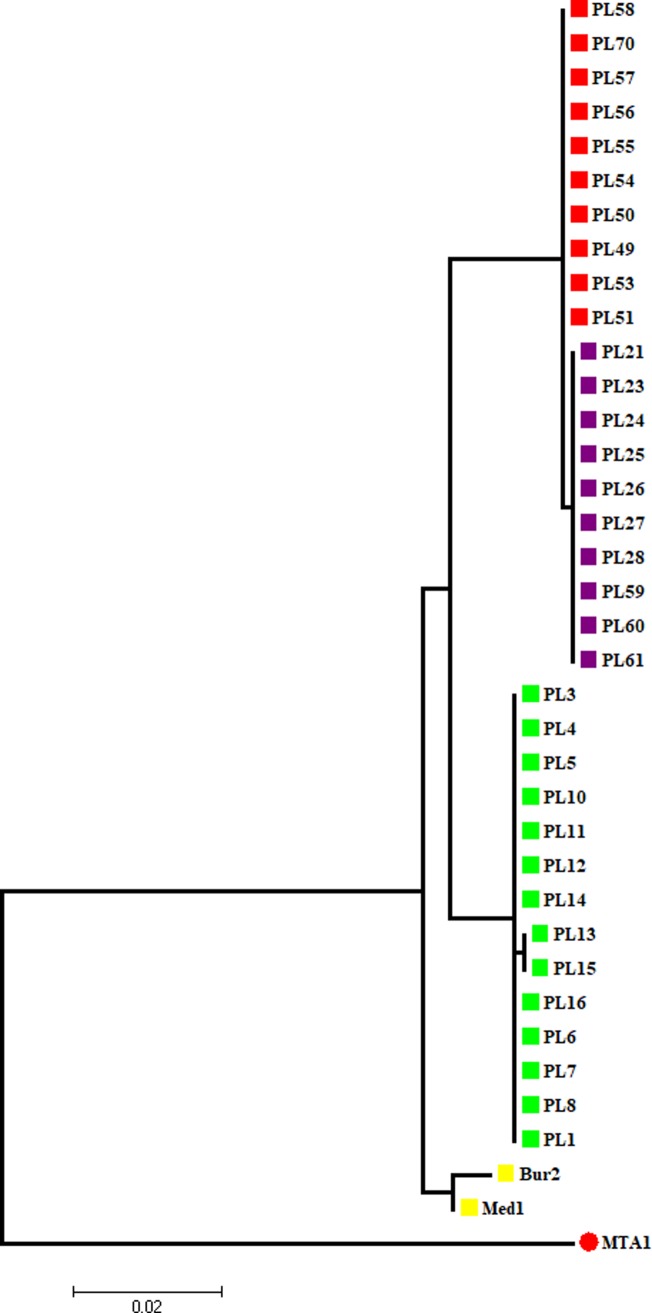
Maximum-likelihood phylogenetic tree based on the ND4 mitochondrial marker. The tree is rooted with *P*. *muralis*. The tree is drawn to scale, with branch lengths measured in the number of substitutions per site. Red squares: Azil; Purple squares: Vaychis; Green squares Foix; Yellow squares: Iberian *P*. *liolepis*; Red circle: outgroup.

Lizards from the three populations were closely related and clearly separated from Iberian *P*. *liolepis* (Fig 6). Individuals from the highland population (Vaychis) cluster within one of the lowland populations and, hence, our sampling does not confound elevation with genetic relatedness. The GenBank accession numbers are included in S7 Table.

## Discussion

Our work showed that *P*. *liolepis* replicates the same pattern of morphological variation with elevation found in closely related species within the genus *Podarcis*, but not in other lizards. We also described temporal and spatial variation in lizard phenotypes. Dorsal and ventral coloration displayed seasonal and interpopulational variation. In addition, we found seasonal variation in the chemical composition of femoral secretions in a temperate continental lizard species and also demonstrated interpopulational variation in chemical profiles.

### Morphology

Highland *P*. *liolepis* were larger, had longer heads and were also more robust than lowland ones. This result agrees with our previous work where *P*. *guadarramae* populations followed Bergmann´s rule[65] (i.e. larger body size in colder environments). It is worth noting that *P*. *liolepis* and *P*. *guadarramae* belong to different lineages recently recognized as distinct species and they inhabit different climatic areas within the Mediterranean region [68, 92]. Thus, deviations from the reversed Bergmann´s clines, as opposed to the vast majority of squamates [23], might be the general trend in this species complex. In addition, highland *P*. *liolepis* had more femoral pores than those from the lowlands, a result also found in *P*. *guadarramae* [57]. It is assumed that femoral pore number reflects an investment in the use of chemical communication [93–94]. In the context of communication optimization, selection may adjust femoral pore number depending on the influence of the physical conditions (e.g. chemical affinity) of the substrate and climatic conditions (e.g. temperature and humidity) on signal detectability and persistence [53, 94–95]. Nonetheless, a recent study found little support for the hypothesis that the diversification of femoral pore number across lacertid lizards co-vary with climatic conditions [96].

### Diversity of compounds in femoral secretions

Lipophilic compounds were highly abundant in *P*. *liolepis* secretions (S6 Table) and showed substantial difference between populations. Strong differences among populations in the lipid composition of femoral secretions are found in insular lacertids such as *Podarcis lilfordi* [58] and *P*. *gaigeae* [56]. Even a recent paper of Ibáñez et al. [60] showed contrasted chemical profiles between marine iguanas in different islands and islets in the Galapagos archipelago. This large difference is expected given the reduced migration and, hence, genetic isolation of insular populations. However, the finding that genetically and geographically close *P*. *liolepis* show high differences in chemical profiles confirm previous results within the *P*. *hispanicus* species complex where this pattern is found even in lizards belonging to the same genetic population [57]. This suggests that plasticity might drive differences in chemical profiles, as it is known for reproductive investment and body size in *P*. *guadarramae* [65–97]. It is worth noting that *P*. *liolepis* from the Columbretes archipelago produce much less diverse femoral gland secretions (47 compounds) [98]. Part of this variation can be attributed to the exclusive compounds found each season in our study. However, the climate of this archipelago is considerably more xeric than in the Pyrenees and it has been suggested that species inhabiting more mesic environment have a higher chemical richness [99].

The seasonal differences found in this work are in line with the scarce scientific literature on seasonal variation in chemical signals of lizards [55, 61]. Specifically, we found that the most abundant compounds, squalene and cholesterol, showed higher levels in the non-reproductive season in both populations. An increase in cholesterol levels in the non-reproductive season was also found in *Gallotia galloti* lizards [61]. Cholesterol has been proposed as a protector of other compounds with potential pheromone activity, lacking a signaling function [96]. Thus, why would lizards increase its levels outside of the reproductive season? One possibility is that chemical signals are not restricted to sexual interactions. Indeed, MacGregor et al. [59] suggested that femoral gland secretions, due to their chemical complexity, may be better suited than any other cue for use in individual recognition because a very high level of specificity is possible. On the other hand, the high diversity of chemicals found in *P*. *liolepis* secretions (142 compounds) fits well to the multiple-message hypothesis which posits that animals emitting rich chemical signals probably have the potential to convey more elaborate messages [99–101]. Indeed, García-Roa et al. [61] showed that another lacertid lizard (*G*. *galloti*) expresses chemical compounds in femoral gland secretions during the non-reproductive season, as we have shown in this work, pointing out the potential role of chemical signals in interactions not only related to reproduction.

Several factors may be responsible for the observed divergence in chemical signals between populations and seasons. For example, some compounds must be acquired through diet [99] and therefore, changes in diet might produce a higher or lesser degree of their expression in scent marks [102–103]. On the other hand, Baeckens et al. [104] provide strong evidence for a significant relationship among species between chemical signal design and prevailing environmental conditions, which may result from differential selection on signaling efficacy.

### Ventral coloration

Lowland lizards were brighter than highland ones in the reproductive season whereas the reversed trend was found in the non-reproductive season only for dorsal brightness. Moreover, lowland and the non-reproductive season lizards were redder than highland and the reproductive season ones, respectively. Conspicuous ventral patches of long wavelength colours have an important role as social signals in lizards [64], and more specifically in lacertids [105–108]. Evolutionary divergence in signal characteristics that improve the efficiency of communication might occur if members of related taxa consistently experience different environmental conditions across many generations (e.g. [109]). In this case, habitat openness or vegetation cover affect light conditions, which may exert strong selection on visual signals, such as ventral coloration, to maximize conspicuousness [110–114] and lead to an increase in the signal-to-noise ratio, or intensity of communication by changing the structural properties of signals [115]. Thus, prevailing light conditions may render some ventral coloration variations more effective than others in different habitats [116], but also between reproductive and non-reproductive seasons, as seasonal variation in body coloration may occur in animals with a distinct reproductive season [116–117]

### Dorsal coloration

With respect to dorsal coloration, lowland lizards were brighter than highland ones in the reproductive season whereas the reversed trend was found in the non-reproductive season. This is in accordance with a thermoregulation hypothesis in which the lower temperatures of high altitude sites would favour darker dorsal coloration, as darker reptiles have several thermoregulation advantages such as fast heating rates and higher thermal inertia [118–119]. Hence, lowland lizards might have a higher dorsal reflectance in the reproductive season as the thermal conditions of their habitat are less restrictive than in the highland site. The same reasoning is argued for *P*. *guadarramae* [120] where highland lizards were darker than lowland ones. Alternatively, animals inhabiting high-altitude habitats can be darker which would protect them against higher UV-radiation as was found in the lizard *Psammodromus algirus* [14]. Nonetheless, these hypotheses are not mutually exclusive and could be both responsible for the results presented here. In addition, we found that brownish coloration was higher in the non-reproductive season whereas greenish coloration was higher in the lowland population and the reproductive season. Most evidence shows that dorsal coloration is generally presumed to be an adaptation for crypsis or thermoregulation in diurnal reptiles [121–123] and background matching has been widely documented in reptiles [106, 124–126]. For instance, interpopulation variability in chromatic features of *P*. *muralis* dorsal pattern are probably locally adapted [127]. Seasonal variation in body coloration may also occur in species living in habitats where background colours [128] or the thermal environment [129] change with the time of year. Thus, background matching to predominant environmental colours determined by plant phenology but also the characteristics of the thermal environment might be responsible for these changes in dorsal coloration between seasons and populations.

When we controlled for body size differences, we found contrasting results between dorsal and ventral coloration. Dorsal coloration did not change with body size as thermoregulatory and antipredatory selective pressures, presumably do not differ along ontogeny. However, seasonal differences in ventral reddish coloration disappeared. These results fit into the idea that signaling and concealment functions are partitioned to different body regions in lizards [105].

Although our results are in line with elevational effects, we cannot ascribe to elevation alone the differences found in this work as we did not replicate high and low elevation sites, except for the two lowland populations considered for morphological analyses. Moreover, given the limited sampling of our study we cannot disentangle the causes of variation in *P*. *liolepis* femoral secretion composition. Nonetheless, the results of this correlational study constitute a valuable source of information regarding interpopulational and seasonal variation in lizard phenotypes that can be used to make further predictions and constitute the stepping-stone to experimentally test specific hypotheses.

## Conclusions

Overall, highland lizards were larger, more robust, had longer heads and more femoral pores and had a darker dorsal coloration than lowland ones. We also detected interpopulational and seasonal variation in femoral secretion composition, ventral, and dorsal coloration. There were strong differences in the abundance and richness of chemical compounds. Ventral and dorsal brightness were higher in lowland than in highland lizards in the reproductive season whereas the reversed trend was only found in the non-reproductive season for dorsal brightness. Lowland and the non-reproductive season lizards were redder than highland and the reproductive season ones, respectively. In addition, all lizards had browner dorsal coloration in the non-reproductive season, and lowland lizards were greener in the reproductive season. Hence, our results highlight that high phenotypic variation can even be present in closely related populations. Despite the logistic difficulties, we encourage future research on widespread taxa over different environmental conditions to disentangle the causes of intraspecific and seasonal variation in phenotypes.

## Supporting information

S1 Table Factor loadings for the PCA on ventral coloration of *Podarcis liolepis* lizards. (DOCX)

S2 Table Factor scores for the PCA on ventral coloration of *Podarcis liolepis* lizards. (DOCX)

S3 Table Factor loadings for the PCA on dorsal coloration of *Podarcis liolepis* lizards. (DOCX)

S4 Table Factor scores for the PCA on dorsal coloration of *Podarcis liolepis* lizards. (DOCX)

S5 Table Summary statistics (mean + SE) for ventral and dorsal coloration in *P*. *liolepis* lizards. (DOCX)

S6 Table Lipophilic compounds found in *P*. *liolepis* femoral pore secretions from two populations in the Midi-Pyrenees. (DOCX)

S7 Table GenBank accesión number for the genetic samples used in this study. PL codes refer to *P*.*liolepis* individuals sampled in the Midi-Pyrenees. Med1 and Bur2 (from Medinaceli and Burgos, respectively) and MT1 (*P*. *muralis*) are sequences obtained from GenBank.
(DOCX)
