# Correction: Seasonal and interpopulational phenotypic variation in morphology and sexual signals of *Podarcis liolepis* lizards

**DOI:** 10.1371/journal.pone.0215629

**Published:** 2019-04-15

**Authors:** Jesús Ortega, José Martín, Pierre-André Crochet, Pilar López, Jean Clobert

In S6 Table, the Spanish word “raro” mistakenly appears in the names of several unidentified esters and acids. This word should not be present. Please view the correct S6 Table below.

## Supporting information

S6 Table Lipophilic compounds found in *P*. *liolepis* femoral pore secretions from two populations in the Midi-Pyrenees. (DOC)
