# Supplementary material for: Seasonal and interpopulational phenotypic variation in morphology and sexual signals of Podarcis liolepis lizards
Source: PLoS One. 2019 Mar 15;14(3):e0211686. doi: 10.1371/journal.pone.0211686 (PMC6419997; doi:10.1371/journal.pone.0211686)
Supplement: S1 Table — (DOCX) [file pone.0211686.s001.docx]

| Wavelength | Ventral PC1 | Ventral PC2 | Ventral PC3 |
| --- | --- | --- | --- |
| 300 | -0,85839 | -0,44420 | -0,135783 |
| 305 | -0,87755 | -0,42772 | -0,094133 |
| 310 | -0,87811 | -0,41514 | -0,152303 |
| 315 | -0,87652 | -0,42834 | -0,137444 |
| 320 | -0,85212 | -0,37960 | -0,134229 |
| 325 | -0,87552 | -0,40142 | -0,096255 |
| 330 | -0,88386 | -0,41267 | -0,138668 |
| 335 | -0,88829 | -0,41159 | -0,159307 |
| 340 | -0,91417 | -0,36792 | -0,138535 |
| 345 | -0,83418 | -0,34478 | -0,104044 |
| 350 | -0,91784 | -0,33505 | -0,146150 |
| 355 | -0,91282 | -0,34587 | -0,167211 |
| 360 | -0,93093 | -0,30727 | -0,155981 |
| 365 | -0,92441 | -0,32031 | -0,182039 |
| 370 | -0,91891 | -0,30441 | -0,203843 |
| 375 | -0,93311 | -0,28369 | -0,185030 |
| 380 | -0,93725 | -0,26728 | -0,175815 |
| 385 | -0,94461 | -0,23046 | -0,186413 |
| 390 | -0,93969 | -0,20197 | -0,173427 |
| 395 | -0,94093 | -0,16345 | -0,174454 |
| 400 | -0,94795 | -0,11373 | -0,179753 |
| 405 | -0,94367 | -0,06547 | -0,181633 |
| 410 | -0,94456 | -0,02057 | -0,200791 |
| 415 | -0,92916 | 0,15187 | -0,181102 |
| 420 | -0,79919 | 0,41439 | -0,139286 |
| 425 | -0,87094 | 0,38759 | -0,115355 |
| 430 | -0,90601 | 0,36689 | -0,131307 |
| 435 | -0,85895 | 0,47424 | -0,081439 |
| 440 | -0,81579 | 0,55745 | -0,079447 |
| 445 | -0,76900 | 0,61818 | -0,073864 |
| 450 | -0,81795 | 0,55362 | -0,077610 |
| 455 | -0,81174 | 0,55588 | -0,083440 |
| 460 | -0,78019 | 0,60399 | -0,072167 |
| 465 | -0,81933 | 0,54338 | -0,073468 |
| 470 | -0,82354 | 0,52881 | -0,078385 |
| 475 | -0,77020 | 0,60392 | -0,068864 |
| 480 | -0,64315 | 0,74775 | -0,043507 |
| 485 | -0,52017 | 0,84028 | -0,021954 |
| 490 | -0,59214 | 0,79143 | -0,026444 |
| 495 | -0,69267 | 0,70153 | -0,037680 |
| 500 | -0,73301 | 0,64593 | -0,058228 |
| 505 | -0,74169 | 0,64764 | -0,036138 |
| 510 | -0,71110 | 0,68755 | -0,023644 |
| 515 | -0,69797 | 0,69759 | -0,011485 |
| 520 | -0,69234 | 0,69708 | -0,009108 |
| 525 | -0,59630 | 0,78510 | 0,043521 |
| 530 | -0,55352 | 0,81049 | 0,065907 |
| 535 | -0,66442 | 0,72051 | 0,078329 |
| 540 | -0,62021 | 0,75042 | 0,119752 |
| 545 | -0,76038 | 0,61223 | 0,111686 |
| 550 | -0,87322 | 0,44047 | 0,140523 |
| 555 | -0,94065 | 0,27044 | 0,137880 |
| 560 | -0,96905 | 0,14619 | 0,151044 |
| 565 | -0,93955 | 0,17763 | 0,233857 |
| 570 | -0,93551 | 0,18938 | 0,238865 |
| 575 | -0,95803 | 0,08593 | 0,223347 |
| 580 | -0,97485 | -0,08763 | 0,180974 |
| 585 | -0,96631 | -0,12871 | 0,195299 |
| 590 | -0,95212 | -0,16775 | 0,216574 |
| 595 | -0,94994 | -0,10121 | 0,252005 |
| 600 | -0,93419 | -0,14995 | 0,276581 |
| 605 | -0,93742 | -0,14172 | 0,268025 |
| 610 | -0,95209 | -0,17007 | 0,228309 |
| 615 | -0,93322 | -0,27559 | 0,200943 |
| 620 | -0,92899 | -0,31240 | 0,166917 |
| 625 | -0,93089 | -0,24970 | 0,219779 |
| 630 | -0,93673 | -0,28141 | 0,186350 |
| 635 | -0,93134 | -0,31753 | 0,141537 |
| 640 | -0,91186 | -0,36543 | 0,139131 |
| 645 | -0,91896 | -0,35648 | 0,109025 |
| 650 | -0,92346 | -0,35617 | 0,119465 |
| 655 | -0,90346 | -0,39342 | 0,125344 |
| 660 | -0,91352 | -0,35924 | 0,087851 |
| 665 | -0,89036 | -0,39384 | 0,085263 |
| 670 | -0,88865 | -0,38899 | 0,060691 |
| 675 | -0,89770 | -0,41277 | 0,073188 |
| 680 | -0,90454 | -0,38638 | 0,095323 |
| 685 | -0,88984 | -0,41834 | 0,029195 |
| 690 | -0,89463 | -0,39427 | -0,002826 |
| 695 | -0,86955 | -0,41695 | -0,002506 |
| 700 | -0,87364 | -0,37292 | -0,031975 |
