# Supplementary material for: Seasonal and interpopulational phenotypic variation in morphology and sexual signals of Podarcis liolepis lizards
Source: PLoS One. 2019 Mar 15;14(3):e0211686. doi: 10.1371/journal.pone.0211686 (PMC6419997; doi:10.1371/journal.pone.0211686)
Supplement: S2 Table — (DOCX) [file pone.0211686.s002.docx]

| Ventral-PC1 | Ventral-PC2 | Ventral-PC3 |
| --- | --- | --- |
| 0,21592 | 1,50864 | -0,25138 |
| 0,40837 | 1,21666 | 0,31553 |
| 0,08679 | 1,73479 | 1,65475 |
| 0,57060 | 1,31418 | -0,68461 |
| 0,16880 | 0,80889 | -0,04304 |
| 0,52732 | 0,28114 | -0,01326 |
| 0,17030 | 1,12909 | -0,67058 |
| 0,62303 | 0,48958 | -0,03665 |
| 0,19328 | 1,12934 | 1,91813 |
| -0,93039 | -0,20955 | -0,32745 |
| -1,02577 | -0,79493 | -0,40090 |
| -1,00671 | -1,71207 | 0,76391 |
| -1,15652 | -1,82715 | 0,87090 |
| -0,85682 | -2,40026 | -0,96989 |
| -2,17485 | -0,46604 | -0,62769 |
| -2,40256 | 0,13656 | 0,43096 |
| -2,27783 | 0,09299 | -0,95781 |
| -2,61446 | -0,13892 | -0,54136 |
| 0,35778 | 1,17819 | -1,09911 |
| 0,60745 | -1,90391 | -1,07513 |
| 1,44396 | -1,32179 | 0,04170 |
| 2,03822 | -1,48961 | -1,09248 |
| 0,55217 | 1,15672 | -0,71985 |
| 1,08011 | -0,19105 | -0,84610 |
| 0,79558 | 0,66193 | -1,05126 |
| 2,30207 | -1,08217 | -1,71212 |
| 1,53389 | -0,16322 | -0,17091 |
| 0,69871 | 0,36546 | 0,61379 |
| 0,97923 | -1,23517 | 2,11816 |
| 0,31548 | 0,78940 | 0,59636 |
| 0,74128 | -1,27060 | 2,28621 |
| 0,19601 | 0,88880 | 0,90024 |
| 0,72659 | -0,46877 | 0,04979 |
| 0,32999 | -0,39384 | 1,52826 |
| 0,32159 | -0,77049 | 1,24774 |
| 0,01695 | 1,12298 | 0,18093 |
| -0,13315 | 0,70950 | 0,39924 |
| -0,08021 | 0,46690 | -0,85502 |
| 0,08231 | 0,65961 | -0,71615 |
| -0,35229 | 0,78023 | 0,11213 |
| 0,15050 | 0,62722 | -0,91434 |
| -0,27559 | 0,77632 | 1,67263 |
| -0,45878 | 0,74595 | -0,77195 |
| 0,18596 | -1,15173 | 1,46029 |
| -0,47840 | 0,16119 | -0,50470 |
| -0,45849 | 0,06113 | -1,32122 |
| -0,57514 | -0,17588 | 1,43148 |
| 0,07180 | -1,08629 | -1,14346 |
| -0,39210 | -0,28286 | -0,98752 |
| -0,50178 | -0,09034 | -0,05992 |
| -0,34021 | -0,36672 | -0,02725 |
