# Supplementary material for: Seasonal and interpopulational phenotypic variation in morphology and sexual signals of Podarcis liolepis lizards
Source: PLoS One. 2019 Mar 15;14(3):e0211686. doi: 10.1371/journal.pone.0211686 (PMC6419997; doi:10.1371/journal.pone.0211686)
Supplement: S3 Table — (DOCX) [file pone.0211686.s003.docx]

| Wavelength | Dorsal-PC1 | Dorsal-PC2 | Dorsal PC3 |
| --- | --- | --- | --- |
| 300 | -0,85593 | -0,504590 | -0,005631 |
| 305 | -0,88908 | -0,434766 | 0,003472 |
| 310 | -0,90529 | -0,412518 | 0,027183 |
| 315 | -0,88999 | -0,431492 | 0,058990 |
| 320 | -0,88893 | -0,278624 | 0,231629 |
| 325 | -0,86864 | -0,470584 | -0,018204 |
| 330 | -0,90217 | -0,399742 | 0,087323 |
| 335 | -0,90138 | -0,412275 | 0,079419 |
| 340 | -0,91567 | -0,385502 | 0,051508 |
| 345 | -0,87093 | -0,235925 | 0,295189 |
| 350 | -0,92645 | -0,362514 | 0,032729 |
| 355 | -0,90982 | -0,400877 | -0,008204 |
| 360 | -0,94060 | -0,301070 | 0,109114 |
| 365 | -0,92858 | -0,336970 | 0,128345 |
| 370 | -0,95549 | -0,254778 | 0,068347 |
| 375 | -0,95023 | -0,282065 | 0,101845 |
| 380 | -0,94302 | -0,318168 | 0,031653 |
| 385 | -0,95364 | -0,265506 | 0,070279 |
| 390 | -0,97312 | -0,192054 | 0,061198 |
| 395 | -0,97788 | -0,166872 | 0,065869 |
| 400 | -0,98339 | -0,104429 | 0,057111 |
| 405 | -0,98403 | -0,096433 | 0,088664 |
| 410 | -0,98043 | -0,122942 | 0,073815 |
| 415 | -0,98600 | 0,066421 | 0,078385 |
| 420 | -0,86968 | 0,447802 | 0,178832 |
| 425 | -0,94522 | 0,281695 | 0,108949 |
| 430 | -0,97462 | 0,130142 | 0,041792 |
| 435 | -0,96022 | 0,224568 | 0,046223 |
| 440 | -0,93163 | 0,326106 | 0,047765 |
| 445 | -0,90097 | 0,410257 | 0,077148 |
| 450 | -0,95223 | 0,264704 | 0,037572 |
| 455 | -0,96035 | 0,244669 | 0,052112 |
| 460 | -0,95140 | 0,281922 | 0,076689 |
| 465 | -0,97613 | 0,172122 | 0,012537 |
| 470 | -0,98543 | 0,130318 | -0,024020 |
| 475 | -0,96983 | 0,211817 | -0,036063 |
| 480 | -0,83662 | 0,537816 | 0,055775 |
| 485 | -0,60405 | 0,784989 | 0,115128 |
| 490 | -0,74056 | 0,666537 | 0,049663 |
| 495 | -0,89161 | 0,442787 | -0,003069 |
| 500 | -0,93894 | 0,329212 | -0,019672 |
| 505 | -0,93815 | 0,331315 | -0,049156 |
| 510 | -0,90287 | 0,417540 | -0,012719 |
| 515 | -0,90327 | 0,416312 | -0,020953 |
| 520 | -0,87101 | 0,474953 | -0,021909 |
| 525 | -0,74940 | 0,653671 | 0,035837 |
| 530 | -0,67186 | 0,732176 | 0,053731 |
| 535 | -0,79749 | 0,596807 | 0,017524 |
| 540 | -0,75852 | 0,646623 | 0,002736 |
| 545 | -0,86965 | 0,486293 | -0,032331 |
| 550 | -0,94687 | 0,275719 | -0,101467 |
| 555 | -0,97323 | 0,159384 | -0,105800 |
| 560 | -0,98897 | 0,073444 | -0,078319 |
| 565 | -0,97879 | 0,142900 | -0,079361 |
| 570 | -0,96088 | 0,243770 | -0,066854 |
| 575 | -0,96912 | 0,207698 | -0,070180 |
| 580 | -0,99048 | 0,005887 | -0,083918 |
| 585 | -0,98876 | -0,027695 | -0,129016 |
| 590 | -0,98037 | -0,090748 | -0,097579 |
| 595 | -0,98461 | 0,062228 | -0,127351 |
| 600 | -0,98265 | 0,052147 | -0,128623 |
| 605 | -0,97900 | 0,142062 | -0,089412 |
| 610 | -0,98222 | 0,044019 | -0,140022 |
| 615 | -0,98122 | -0,115966 | -0,123530 |
| 620 | -0,96737 | -0,148361 | -0,165591 |
| 625 | -0,97892 | -0,052096 | -0,160897 |
| 630 | -0,98695 | -0,078642 | -0,104847 |
| 635 | -0,97997 | -0,133761 | -0,113780 |
| 640 | -0,94164 | -0,281378 | -0,105752 |
| 645 | -0,96218 | -0,229715 | -0,106946 |
| 650 | -0,97965 | -0,164959 | -0,049092 |
| 655 | -0,95885 | -0,258423 | -0,066237 |
| 660 | -0,96033 | -0,238637 | -0,074698 |
| 665 | -0,91765 | -0,363249 | -0,111272 |
| 670 | -0,93996 | -0,286167 | -0,089938 |
| 675 | -0,95940 | -0,265482 | -0,012500 |
| 680 | -0,96995 | -0,213645 | -0,016227 |
| 685 | -0,94236 | -0,315429 | -0,020098 |
| 690 | -0,94621 | -0,243799 | 0,039164 |
| 695 | -0,93834 | -0,266129 | 0,080722 |
| 700 | -0,93779 | -0,188668 | 0,140807 |
