# Supplementary material for: Seasonal and interpopulational phenotypic variation in morphology and sexual signals of Podarcis liolepis lizards
Source: PLoS One. 2019 Mar 15;14(3):e0211686. doi: 10.1371/journal.pone.0211686 (PMC6419997; doi:10.1371/journal.pone.0211686)
Supplement: S4 Table — (DOCX) [file pone.0211686.s004.docx]

| Dorsal-PC1 | Dorsal-PC2 | Dorsal-PC3 |
| --- | --- | --- |
| -0,94904 | 3,54833 | -3,88776 |
| 0,12785 | 1,84665 | 1,23040 |
| 0,01916 | 2,04125 | 0,42109 |
| 0,15024 | 1,80897 | 1,12522 |
| 0,15939 | 1,77487 | 1,01281 |
| 0,07468 | 1,95994 | 0,95666 |
| -0,02013 | 2,10758 | 1,04109 |
| 0,04494 | 1,86078 | 0,87351 |
| 0,06413 | 0,50665 | 0,14951 |
| -0,61112 | -0,78860 | 1,37389 |
| -1,17400 | -0,27435 | 0,38765 |
| -1,76191 | -0,04493 | -0,08767 |
| -2,04239 | 0,23976 | -0,02099 |
| -1,68119 | -0,75313 | 1,22752 |
| -2,00360 | -0,59993 | 0,51402 |
| -1,96256 | -0,61226 | 0,91362 |
| -2,23695 | -0,32512 | 0,94802 |
| -2,14295 | -0,55371 | 1,40661 |
| 0,78492 | -0,27065 | 0,75221 |
| -0,21373 | -0,78740 | 0,09309 |
| 1,10676 | -0,41627 | 0,27101 |
| 1,26567 | -0,44021 | 0,72020 |
| 0,80543 | 0,01027 | 0,30775 |
| 1,08988 | -0,68067 | 1,40505 |
| 0,78818 | -0,45997 | -0,24413 |
| 1,63257 | -0,43616 | 0,91799 |
| 1,53152 | -0,41472 | 0,53244 |
| 1,28071 | -0,19575 | 0,07301 |
| 0,81450 | 0,05489 | -0,55320 |
| 1,03095 | -0,48643 | 0,16638 |
| 0,87413 | -0,34539 | -0,60262 |
| 0,79747 | -0,25262 | 0,06217 |
| 0,57423 | 0,02289 | -0,29946 |
| 0,62100 | -0,17392 | -0,98581 |
| 0,69942 | -0,38097 | 0,12647 |
| 0,42106 | 0,16133 | -0,71991 |
| 0,48971 | 0,01691 | -0,69923 |
| 0,63208 | -0,37066 | 0,07300 |
| 0,52118 | -0,42858 | 0,25523 |
| 0,30051 | 0,06496 | -1,22706 |
| 0,65420 | -0,55517 | 0,13591 |
| 0,15456 | -0,15704 | -0,73182 |
| 0,09136 | -0,56529 | -0,42848 |
| -0,22806 | -0,35174 | -1,06110 |
| -0,07483 | -0,82996 | -0,38735 |
| -0,42128 | -0,63455 | -0,97449 |
| -0,30677 | -1,04587 | -1,58150 |
| -0,52787 | -0,78849 | -1,30630 |
| -0,33136 | -0,90459 | -0,94278 |
| -0,50766 | -0,81493 | -1,29696 |
| -0,40497 | -0,88600 | -1,43488 |
