# Supplementary material for: Seasonal and interpopulational phenotypic variation in morphology and sexual signals of Podarcis liolepis lizards
Source: PLoS One. 2019 Mar 15;14(3):e0211686. doi: 10.1371/journal.pone.0211686 (PMC6419997; doi:10.1371/journal.pone.0211686)
Supplement: S5 Table — (DOCX) [file pone.0211686.s005.docx]

|  |  |  |  | **Ventral coloration** | | | | | | | | | **Dorsal coloration** | | | | | | | | |
| --- | --- | --- | --- | --- | --- | --- | --- | --- | --- | --- | --- | --- | --- | --- | --- | --- | --- | --- | --- | --- | --- |
| **Population** | **Season** | **Sex** | **N** | **PC1** | | | **PC2** | | | **PC3** | | | **PC1** | | | **PC2** | | | **PC3** | | |
| FOIX | R | M | 11 | -0,80 | + | 0,24 | 0,10 | + | 0,31 | 0,23 | + | 0,29 | -1,13 | + | 0,20 | 0,70 | + | 0,26 | 0,31 | + | 0,26 |
| FOIX | R | F | 7 | -0,38 | + | 0,31 | 0,18 | + | 0,39 | -0,30 | + | 0,36 | -0,50 | + | 0,25 | 0,86 | + | 0,33 | 0,88 | + | 0,32 |
| FOIX | NR | M | 9 | 0,77 | + | 0,27 | -0,08 | + | 0,34 | 0,39 | + | 0,32 | 0,91 | + | 0,22 | -0,24 | + | 0,29 | -0,03 | + | 0,29 |
| FOIX | NR | F | 6 | 0,13 | + | 0,33 | 0,19 | + | 0,42 | 0,51 | + | 0,39 | 0,67 | + | 0,27 | -0,17 | + | 0,35 | -0,44 | + | 0,35 |
| Vaychis | R | M | 6 | 1,09 | + | 0,33 | -0,51 | + | 0,42 | -0,79 | + | 0,39 | 0,81 | + | 0,27 | -0,46 | + | 0,35 | 0,43 | + | 0,35 |
| Vaychis | R | F | 1 | 0,36 | + | 0,81 | 1,18 | + | 1,03 | -1,10 | + | 0,96 | 0,78 | + | 0,67 | -0,27 | + | 0,86 | 0,75 | + | 0,86 |
| Vaychis | NR | M | 7 | -0,34 | + | 0,31 | 0,09 | + | 0,39 | 0,09 | + | 0,36 | -0,12 | + | 0,25 | -0,74 | + | 0,33 | -0,89 | + | 0,32 |
| Vaychis | NR | F | 4 | -0,16 | + | 0,41 | -0,36 | + | 0,52 | -0,44 | + | 0,48 | -0,27 | + | 0,33 | -0,59 | + | 0,43 | -0,94 | + | 0,43 |
