# Supplementary material for: Seasonal and interpopulational phenotypic variation in morphology and sexual signals of Podarcis liolepis lizards
Source: PLoS One. 2019 Mar 15;14(3):e0211686. doi: 10.1371/journal.pone.0211686 (PMC6419997; doi:10.1371/journal.pone.0211686)
Supplement: S6 Table — (DOCX) [file pone.0211686.s006.docx]

|  | |  | | | Spring  (*n = 15*) | | | | | | | | | | | Autumn  (*n = 17*) | | | | | | | |  |
| --- | --- | --- | --- | --- | --- | --- | --- | --- | --- | --- | --- | --- | --- | --- | --- | --- | --- | --- | --- | --- | --- | --- | --- | --- |
|  | |  | | | Vaychis  (*n = 5*) | | | | Foix  (*n = 10*) | | | | | | | | Vaychis  (*n = 8*) | | | | Foix  (*n = 9*) | | | |
| RT | Compound | |  | | |  |  | |  | |  | |  | |  | | |  |  |  | |  |  |  |
|  |  | |  | | |  |  | |  | |  | |  | |  | | |  |  |  | |  |  |  |
| Carboxilic acids and their esters | | | |  | | | |  |  |  | |  | |  |  |  |  |  |  |  |  |  |  |  |
| 21,14 | Tetradecanoic acid | | 0.01 | | | + | 0.02 | |  | | - | |  | |  | | | - |  |  | | - |  |  |
| 29,41 | Tetradecanoic acid, 1-methylethyl ester | |  | | | - |  | |  | | - | |  | | 0.28 | | | + | 0.38 |  | | - |  |  |
| 31,51 | Hexadecanoic acid, methyl ester | |  | | | - |  | |  | | - | |  | | 0.13 | | | + | 0.11 |  | | - |  |  |
| 32,89 | Hexadecanoic acid, ethyl ester | |  | | | - |  | | 0.06 | | + | | 0.12 | | 0.41 | | | + | 0.42 |  | | - |  |  |
| 33,06 | Hexadecanoic acid | | 0.33 | | | + | 0.35 | | 0.08 | | + | | 0.10 | |  | | | - |  |  | | - |  |  |
| 33,41 | Hexadecanoic acid, 1-methylethyl ester | | 0.05 | | | + | 0.11 | | 0.04 | | + | | 0.07 | | 3.59 | | | + | 0.93 | 0.77 | | + | 1.76 |  |
| 34,82 | 9,12-octadecadienoic acid, methyl ester | | 0.01 | | | + | 0.02 | |  | | - | |  | |  | | | - |  |  | | - |  |  |
| 34,89 | 9-octadecenoic acid (z)-, methyl ester | | 0.01 | | | + | 0.02 | |  | | - | |  | |  | | | - |  |  | | - |  |  |
| 35,12 | Octadecanoic acid, methyl ester | |  | | | - |  | |  | | - | |  | | 0.13 | | | + | 0.17 |  | | - |  |  |
| 35,93 | 9,12-octadecadienoic acid, ethyl ester | | 0.02 | | | + | 0.03 | | 0.04 | | + | | 0.07 | | 0.59 | | | + | 0.35 | 0.04 | | + | 0.13 |  |
| 36,01 | 9-octadecenoic acid ethyl ester | | 0.01 | | | + | 0.01 | | 0.03 | | + | | 0.04 | | 0.88 | | | + | 0.43 | 0.04 | | + | 0.11 |  |
| 36.44 | Octadecanoic acid. ethyl ester | | 0.00 | | | + | 0.01 | | 0.02 | | + | | 0.02 | |  | | | - |  |  | | - |  |  |
| 37.12 | Octadecanoic acid | | 0.01 | | | + | 0.02 | |  | | - | |  | |  | | | - |  |  | | - |  |  |
| 37.43 | 5.8.11.14-eicosatetraenoic acid. | | 0.00 | | | + | 0.01 | |  | | - | |  | |  | | | - |  |  | | - |  |  |
| 37.61 | 5.8.11.14-eicosatetraenoic acid. methyl ester | | 0.01 | | | + | 0.01 | |  | | - | |  | |  | | | - |  |  | | - |  |  |
| 38.6 | 5.8.11.14-eicosatetraenoic acid. ethyl ester | | 0.00 | | | + | 0.01 | | 0.02 | | + | | 0.02 | |  | | | - |  |  | | - |  |  |
| Steroids | | | |  | | | |  |  |  | |  | |  |  |  |  |  |  |  |  |  |  |  |
| 45.17 | Unidentified steroid (145, 213, 247, 255, 363, 368, 395) | |  | | | - |  | |  | | - | |  | |  | | | - |  | 1.82 | | + | 1.16 |  |
| 45.37 | Unidentified steroid (145, 255, 326, 352, 369, 388) | |  | | | - |  | | 0.06 | | + | | 0.08 | |  | | | - |  |  | | - |  |  |
| 46.07 | Unidentified steroid (199, 253, 352, 367) | | 0.04 | | | + | 0.07 | | 0.02 | | + | | 0.04 | |  | | | - |  |  | | - |  |  |
| 46.31 | Cholest-2-ene | |  | | | - |  | |  | | - | |  | |  | | | - |  | 0.09 | | + | 0.16 |  |
| 46.71 | Cholesta-2,4-diene | | 0.02 | | | + | 0.03 | |  | | - | |  | |  | | | - |  | 1.96 | | + | 1.32 |  |
| 46.89 | Unidentified steroid (141, 156, 350, 365) | | 0.67 | | | + | 0.48 | | 0.22 | | + | | 0.08 | |  | | | - |  |  | | - |  |  |
| 46.89 | Unidentified cholesta-2,4-diene, Derivative | |  | | | - |  | |  | | - | |  | |  | | | - |  | 0.44 | | + | 0.33 |  |
| 47.1 | Cholesta-4,6-dien-3-ol | | 0.10 | | | + | 0.10 | | 0.19 | | + | | 0.08 | | 2.58 | | | + | 1.65 | 4.23 | | + | 2.58 |  |
| 47.2 | Cholesta-3,5-diene | | 0.20 | | | + | 0.15 | | 0.10 | | + | | 0.08 | | 1.41 | | | + | 0.41 | 6.83 | | + | 3.73 |  |
| 47.34 | Unidentified steroid (155, 197, 251, 349, 384) | |  | | | - |  | |  | | - | |  | |  | | | - |  | 0.80 | | + | 0.93 |  |
| 47.54 | Unidentified steroid (105, 155, 197, 251, 350, 365) | | 3.21 | | | + | 1.62 | | 1.80 | | + | | 0.94 | |  | | | - |  | 0.40 | | + | 0.41 |  |
| 47.78 | Cholesta-5,7,9 (11)-trien-3-ol, | | 3.28 | | | + | 1.59 | | 0.20 | | + | | 0.41 | |  | | | - |  | 0.34 | | + | 0.35 |  |
| 47.89 | Unidentified steroid (195, 209, 251, 350, 365, 387) | | 0.38 | | | + | 0.22 | |  | | - | |  | |  | | | - |  | 0.32 | | + | 0.31 |  |
| 47.99 | Cholesta-5,7,9 (11)-trien-3-ol, Derivative | |  | | | - |  | | 0.52 | | + | | 0.39 | |  | | | - |  |  | | - |  |  |
| 48.04 | Unidentified steroid (183, 195, 210, 237, 350, 365) | | 0.84 | | | + | 0.40 | |  | | - | |  | |  | | | - |  |  | | - |  |  |
| 48.12 | Unidentified cholesta-4,6-dien-3-ol, methyl derivative | |  | | | - |  | |  | | - | |  | | 0.12 | | | + | 0.27 | 0.17 | | + | 0.22 |  |
| 48.34 | Unidentified steroid (199, 253, 367, 379, 394) | |  | | | - |  | |  | | - | |  | |  | | | - |  | 1.08 | | + | 0.75 |  |
| 48.47 | Unidentified steroid (141, 156, 209, 350, 365) | | 1.48 | | | + | 0.77 | | 0.88 | | + | | 0.35 | |  | | | - |  |  | | - |  |  |
| 48.74 | Unidentified steroid (181, 193, 235, 349, 365) | | 0.05 | | | + | 0.06 | |  | | - | |  | |  | | | - |  |  | | - |  |  |
| 48.83 | Unidentified steroid (215, 233, 257, 344, 383, 401, 416) | |  | | | - |  | |  | | - | |  | |  | | | - |  | 0.40 | | + | 0.28 |  |
| 48.9 | Unidentified steroid (105, 155, 197, 251, 364, 379) | | 0.11 | | | + | 0.03 | | 0.19 | | + | | 0.06 | | 0.26 | | | + | 0.26 | 0.57 | | + | 0.50 |  |
| 49.04 | Unidentified steroid (195, 209, 235, 362, 377) | | 0.08 | | | + | 0.08 | |  | | - | |  | |  | | | - |  |  | | - |  |  |
| 49.15 | Unidentified steroid (195, 209, 235, 364, 379) | | 0.11 | | | + | 0.09 | |  | | - | |  | |  | | | - |  |  | | - |  |  |
| 49.25 | Cholest-5-ene, 3-methoxy | | 2.39 | | | + | 1.77 | | 7.48 | | + | | 2.66 | | 0.24 | | | + | 0.27 | 0.81 | | + | 0.47 |  |
| 49.43 | Cholestane, 3-methoxy | | 0.27 | | | + | 0.17 | | 0.37 | | + | | 0.17 | | 0.24 | | | + | 0.12 | 0.17 | | + | 0.16 |  |
| 49.47 | Stigmastan-3,5-diene | |  | | | - |  | |  | | - | |  | |  | | | - |  | 0.40 | | + | 0.37 |  |
| 49.77 | Unidentified (197, 209, 224, 251, 365, 379) | | 0.08 | | | + | 0.08 | |  | | - | |  | |  | | | - |  |  | | - |  |  |
| 49.98 | Unidentified (105, 155, 197, 251, 355, 378, 393) | | 0.13 | | | + | 0.10 | | 0.22 | | + | | 0.12 | | 1.62 | | | + | 0.76 | 3.11 | | + | 1.20 |  |
| 50.22 | Cholesterol | | 6.94 | | | + | 1.52 | | 8.77 | | + | | 2.45 | | 12.53 | | | + | 5.95 | 20.36 | | + | 9.66 |  |
| 50.25 | Unidentified steroid (143, 158, 195, 253, 378, 394) | |  | | | - |  | |  | | - | |  | |  | | | - |  | 0.68 | | + | 0.58 |  |
| 50.35 | Unidentified steroid (183, 195, 210, 237, 327, 355, 378, 401) | |  | | | - |  | |  | | - | |  | | 0.41 | | | + | 0.22 | 0.36 | | + | 0.32 |  |
| 50.58 | Unidentified methyl derivative steroid (215, 231, 327, 384, 400, 415) | | 0.38 | | | + | 0.28 | | 1.56 | | + | | 0.67 | | 0.39 | | | + | 0.18 | 0.48 | | + | 0.20 |  |
| 50.7 | Unidentified steroid (221, 235, 361, 377, 401) | |  | | | - |  | |  | | - | |  | |  | | | - |  | 0.25 | | + | 0.22 |  |
| 50.77 | Cholestane, 3-methoxy-, methoxy | | 0.97 | | | + | 0.73 | | 1.52 | | + | | 1.22 | |  | | | - |  |  | | - |  |  |
| 50.77 | 9-hexadecenoic acid, hexadecyl ester | |  | | | - |  | |  | | - | |  | | 0.28 | | | + | 0.25 |  | | - |  |  |
| 51.02 | Unidentified steroid (199, 211, 226, 253, 326, 352, 367, 385) | | 0.30 | | | + | 0.34 | |  | | - | |  | |  | | | - |  |  | | - |  |  |
| 51.61 | B-sitosterol methyl ether | | 0.51 | | | + | 0.23 | | 0.98 | | + | | 0.38 | |  | | | - |  | 0.47 | | + | 0.19 |  |
| 51.72 | Cholest-4-en-3-one | | 0.15 | | | + | 0.16 | |  | | - | |  | | 0.89 | | | + | 1.14 | 0.34 | | + | 0.12 |  |
| 51.79 | Cholestane, 3-ethoxy-, methoxy | | 6.15 | | | + | 1.74 | | 9.76 | | + | | 3.68 | | 2.51 | | | + | 1.57 | 5.51 | | + | 1.93 |  |
| 51.97 | Unidentified steroid (211, 229, 316, 331, 366, 399) | |  | | | - |  | | 0.16 | | + | | 0.20 | |  | | | - |  |  | | - |  |  |
| 52.08 | Unidentified steroid (136, 227, 269, 339, 366, 383, 401) | |  | | | - |  | | 0.43 | | + | | 0.29 | |  | | | - |  |  | | - |  |  |
| 52.24 | Cholesta-4,6-dien-3-one | | 3.84 | | | + | 3.47 | | 0.42 | | + | | 0.28 | | 1.00 | | | + | 0.91 | 1.99 | | + | 1.03 |  |
| 52.38 | Cholest-7-en-3-ol, acetate | | 0.34 | | | + | 0.17 | | 0.41 | | + | | 0.45 | |  | | | - |  |  | | - |  |  |
| 52.61 | Unidentified steroid (211, 267, 366, 379) | | 0.19 | | | + | 0.05 | |  | | - | |  | |  | | | - |  |  | | - |  |  |
| 52.84 | Unidentified steroid (214, 253, 267, 280, 296, 366, 381) | | 4.87 | | | + | 1.49 | | 5.45 | | + | | 3.85 | |  | | | - |  | 1.05 | | + | 1.02 |  |
| 52.85 | Unidentified sitosterol, derivative (438) | |  | | | - |  | |  | | - | |  | | 0.60 | | | + | 0.25 |  | | - |  |  |
| 52.92 | Unidentified steroid (231, 267, 371, 382, 400, 415) | |  | | | - |  | |  | | - | |  | |  | | | - |  | 0.34 | | + | 0.46 |  |
| 53.54 | Unidentified steroid (215, 227, 269, 353, 397) | | 0.03 | | | + | 0.07 | |  | | - | |  | |  | | | - |  |  | | - |  |  |
| 54.18 | Unidentified steroid (214, 253, 267, 280, 296, 380, 395) | | 0.24 | | | + | 0.16 | | 0.16 | | + | | 0.15 | |  | | | - |  |  | | - |  |  |
| 55.7 | Unidentified steroid (145, 283, 354, 369, 387, 401, 416) | |  | | | - |  | | 0.18 | | + | | 0.15 | |  | | | - |  |  | | - |  |  |
| Alcohols | | | |  | | | |  |  |  | |  | |  |  |  |  |  |  |  |  |  |  |  |
| 14.47 | Dodecanol | | 0.02 | | | + | 0.01 | |  | | - | |  | |  | | | - |  |  | | - |  |  |
| 23.46 | Tetradecanol | |  | | | - |  | |  | | - | |  | |  | | | - |  | 0.15 | | + | 0.45 |  |
| 29.05 | Pentadecanol | |  | | | - |  | |  | | - | |  | | 0.13 | | | + | 0.11 | 0.17 | | + | 0.37 |  |
| 31.03 | Hexadecanol | | 0.35 | | | + | 0.29 | | 0.12 | | + | | 0.11 | | 0.40 | | | + | 0.49 | 1.22 | | + | 1.00 |  |
| 34.8 | Octadecanol | | 0.23 | | | + | 0.17 | | 0.19 | | + | | 0.17 | |  | | | - |  |  | | - |  |  |
| 36.61 | Unidentified alcohol | |  | | | - |  | |  | | - | |  | |  | | | - |  | 0.60 | | + | 1.01 |  |
| 38.02 | Eicosanol | | 0.04 | | | + | 0.05 | | 0.01 | | + | | 0.03 | |  | | | - |  |  | | - |  |  |
| Waxy esters | | | | | | | |  |  |  | |  | |  |  |  |  |  |  |  |  |  |  |  |
| 49.6 | 9-hexadecenoic acid. tetradecyl ester | |  | | | - |  | |  | | - | |  | | 0.55 | | | + | 0.18 |  | | - |  |  |
| 50.04 | Tetradecanoic acid. hexadecyl ester | | 0.03 | | | + | 0.07 | |  | | - | |  | |  | | | - |  |  | | - |  |  |
| 51.45 | 9-hexadecenoic acid. octadecyl ester | |  | | | - |  | |  | | - | |  | | 0.11 | | | + | 0.15 |  | | - |  |  |
| 51.9 | 9-hexadecenoic acid. eicosyl ester. | |  | | | - |  | |  | | - | |  | | 1.74 | | | + | 0.77 |  | | - |  |  |
| 52.3 | 9-hexadecenoic acid. eicosyl ester. | |  | | | - |  | |  | | - | |  | | 0.30 | | | + | 0.48 |  | | - |  |  |
| 52.31 | Hexadecanoic acid. hexadecyl ester | | 0.44 | | | + | 0.58 | | 0.80 | | + | | 0.47 | | 0.95 | | | + | 0.51 | 0.13 | | + | 0.26 |  |
| 52.72 | 9-hexadecenoic acid. eicosyl ester. | |  | | | - |  | |  | | - | |  | | 1.02 | | | + | 0.29 |  | | - |  |  |
| 53.4 | Ester del hexadecanoic ac. Raro (244, 258) | | 0.19 | | | + | 0.11 | | 0.27 | | + | | 0.10 | |  | | | - |  |  | | - |  |  |
| 53.9 | 9-hexadecenoic acid. eicosyl ester | | 0.02 | | | + | 0.05 | |  | | - | |  | | 0.23 | | | + | 0.20 |  | | - |  |  |
| 54.17 | 9-hexadecenoic acid. eicosyl ester. | |  | | | - |  | |  | | - | |  | | 2.41 | | | + | 0.50 | 0.27 | | + | 0.81 |  |
| 54.4 | 9-octadecenoic acid (z)-. hexadecyl ester | | 1.31 | | | + | 0.39 | | 1.22 | | + | | 0.66 | |  | | | - |  |  | | - |  |  |
| 54.5 | 9-octadecenoic acid xx ester raro (236.264) | | 0.27 | | | + | 0.12 | | 0.37 | | + | | 0.30 | |  | | | - |  |  | | - |  |  |
| 54.62 | Hexadecanoic acid. octadecyl ester | | 4.41 | | | + | 2.72 | | 7.70 | | + | | 4.07 | | 1.19 | | | + | 0.57 | 1.58 | | + | 2.45 |  |
| 55.16 | 9-hexadecenoic acid. eicosyl ester. | |  | | | - |  | |  | | - | |  | | 0.47 | | | + | 0.30 |  | | - |  |  |
| 55.5 | 9-hexadecenoic acid. eicosyl ester. | |  | | | - |  | |  | | - | |  | | 0.49 | | | + | 0.32 |  | | - |  |  |
| 55.75 | 9-octadecenoic acid xx ester raro (264, 284) | | 0.13 | | | + | 0.14 | | 0.70 | | + | | 0.53 | |  | | | - |  |  | | - |  |  |
| 55.99 | Ester del hexadecanoic ac. Raro (244, 257, 272) | | 0.45 | | | + | 0.29 | |  | | - | |  | |  | | | - |  |  | | - |  |  |
| 56.39 | 9-octadecenoic acid (z)-. octadecil ester | | 10.30 | | | + | 5.89 | | 4.17 | | + | | 2.71 | |  | | | - |  |  | | - |  |  |
| 56.43 | 9-hexadecenoic acid. eicosyl ester. | |  | | | - |  | |  | | - | |  | | 0.44 | | | + | 0.34 |  | | - |  |  |
| 56.56 | 9-octadecenoic acid (z)-.nonadecil ester | |  | | | - |  | | 1.00 | | + | | 0.76 | |  | | | - |  |  | | - |  |  |
| 56.8 | 9-hexadecenoic acid. 9-hexadecenyl ester | |  | | | - |  | |  | | - | |  | | 0.77 | | | + | 0.83 |  | | - |  |  |
| 56.84 | 9-octadecenoic acid xx ester raro (257, 264) | | 0.75 | | | + | 0.36 | |  | | - | |  | |  | | | - |  |  | | - |  |  |
| 57.07 | 9-hexadecenoic acid. eicosyl ester. | |  | | | - |  | |  | | - | |  | | 2.41 | | | + | 0.90 | 0.27 | | + | 0.80 |  |
| 57.12 | Hexadecanoic acid. nonadecyl ester | | 0.35 | | | + | 0.12 | | 0.94 | | + | | 0.79 | | 1.70 | | | + | 1.91 | 4.06 | | + | 5.26 |  |
| 57.39 | 9-octadecenoic acid (z)-. eicosyl ester | | 6.71 | | | + | 2.82 | | 4.90 | | + | | 2.02 | |  | | | - |  |  | | - |  |  |
| 57.5 | 9-octadecenoic acid xx ester raro (236, 264) | | 1.40 | | | + | 0.90 | | 0.93 | | + | | 0.48 | |  | | | - |  |  | | - |  |  |
| 57.66 | Hexadecanoic acid. eicosyl ester | | 4.59 | | | + | 2.38 | | 7.89 | | + | | 3.35 | |  | | | - |  |  | | - |  |  |
| 58.27 | 9-octadecenoic acid xx ester raro | | 0.07 | | | + | 0.08 | |  | | - | |  | |  | | | - |  |  | | - |  |  |
| 58.4 | 9-hexadecenoic acid. eicosyl ester. | |  | | | - |  | |  | | - | |  | | 0.93 | | | + | 0.50 |  | | - |  |  |
| 58.58 | Hexadecanoic acid. 2-(octadecyloxy)ethyl ester | | 0.03 | | | + | 0.05 | |  | | - | |  | |  | | | - |  |  | | - |  |  |
| 59.22 | 9-octadecenoic acid (z)-. heneicosyl ester | | 0.28 | | | + | 0.15 | |  | | - | |  | |  | | | - |  |  | | - |  |  |
| 59.54 | Hexadecanoic acid. heneicosyl ester | | 0.29 | | | + | 0.13 | | 0.42 | | + | | 0.22 | |  | | | - |  | 0.30 | | + | 0.48 |  |
| 61.03 | 9-hexadecenoic acid. eicosyl ester. | |  | | | - |  | |  | | - | |  | | 0.78 | | | + | 0.50 | 0.11 | | + | 0.32 |  |
| 61.48 | 9-octadecenoic acid (z)-. docosyl ester | | 7.03 | | | + | 2.15 | | 4.99 | | + | | 1.88 | |  | | | - |  |  | | - |  |  |
| 61.64 | 9-octadecenoic acid xx ester raro (236, 264) | | 1.27 | | | + | 0.63 | | 0.76 | | + | | 0.32 | |  | | | - |  |  | | - |  |  |
| 61.85 | Hexadecanoic acid. docosyl ester | | 3.64 | | | + | 2.01 | | 5.29 | | + | | 1.43 | | 1.10 | | | + | 1.29 | 2.31 | | + | 2.99 |  |
| 62.73 | 9-octadecenoic acid xx ester raro | | 0.05 | | | + | 0.05 | |  | | - | |  | |  | | | - |  |  | | - |  |  |
| 62.93 | 9-hexadecenoic acid. eicosyl ester. | |  | | | - |  | |  | | - | |  | | 0.14 | | | + | 0.24 |  | | - |  |  |
| 63.62 | 9-hexadecenoic acid. eicosyl ester. | |  | | | - |  | |  | | - | |  | | 0.22 | | | + | 0.25 |  | | - |  |  |
| 64.07 | 9-octadecenoic acid xx ester raro (264, 293) | | 0.94 | | | + | 0.73 | |  | | - | |  | |  | | | - |  |  | | - |  |  |
| 64.54 | Ester del hexadecanoic ac. Raro (244, 257, 272) | | 0.30 | | | + | 0.18 | | 0.50 | | + | | 0.16 | |  | | | - |  |  | | - |  |  |
| 66.37 | 9-octadecenoic acid (z)-. 9-octadecenyl ester | | 0.28 | | | + | 0.27 | | 0.33 | | + | | 0.29 | |  | | | - |  |  | | - |  |  |
| 66.68 | 9-hexadecenoic acid. eicosyl ester. | |  | | | - |  | |  | | - | |  | | 1.02 | | | + | 0.57 | 0.12 | | + | 0.37 |  |
| 67.27 | 9-octadecenoic acid tetracosyl ester | | 4.94 | | | + | 0.57 | | 3.67 | | + | | 1.76 | |  | | | - |  |  | | - |  |  |
| 67.54 | 9-octadecenoic acid xx ester raro (236, 264) | | 1.05 | | | + | 0.42 | | 0.77 | | + | | 0.54 | |  | | | - |  |  | | - |  |  |
| 67.81 | Hexadecanoic acid. tetracosyl ester | | 3.29 | | | + | 2.02 | | 5.35 | | + | | 1.58 | | 1.22 | | | + | 0.98 | 1.10 | | + | 1.75 |  |
| 43.51 | 9-octadecenoic acid (z)-. octyl ester | | 0.40 | | | + | 0.14 | |  | | - | |  | |  | | | - |  |  | | - |  |  |
| 44.83 | 9-octadecenoic acid (z)-. nonyl ester | | 0.30 | | | + | 0.09 | |  | | - | |  | |  | | | - |  |  | | - |  |  |
| 45.35 | 9-octadecenoic acid (z)-. decyl ester | | 1.70 | | | + | 1.05 | |  | | - | |  | |  | | | - |  |  | | - |  |  |
| 46.1 | 9-octadecenoic acid (z)-. dodecyl ester | | 0.04 | | | + | 0.07 | |  | | - | |  | |  | | | - |  |  | | - |  |  |
| 51.36 | 9-octadecenoic acid (z)-. tetradecyl ester | | 0.33 | | | + | 0.34 | |  | | - | |  | |  | | | - |  |  | | - |  |  |
| Terpenoids | | | | | | | |  |  |  | |  | |  |  |  |  |  |  |  |  |  |  |  |
| 45.72 | Squalene | | 2.23 | | | + | 1.93 | | 1.41 | | + | | 0.91 | | 44.96 | | | + | 8.51 | 11.12 | | + | 19.42 |  |
| 47.33 | Unidentified terpenoid | | 0.10 | | | + | 0.05 | | 2.44 | | + | | 1.00 | |  | | | - |  |  | | - |  |  |
| 48.75 | Unidentified terpenoid | |  | | | - |  | |  | | - | |  | | 0.18 | | | + | 0.33 |  | | - |  |  |
| 49.57 | Unidentified terpenoid | | 0.38 | | | + | 0.27 | | 0.41 | | + | | 0.40 | | 0.41 | | | + | 0.29 |  | | - |  |  |
| Aldehydes | | | | | | | |  |  |  | |  | |  |  |  |  |  |  |  |  |  |  |  |
| 26.7 | E-14-hexadecenal | |  | | | - |  | |  | | - | |  | | 1.01 | | | + | 0.49 | 13.23 | | + | 11.78 |  |
| 29.36 | Tetradecanal | |  | | | - |  | |  | | - | |  | | 1.41 | | | + | 1.27 | 4.58 | | + | 2.08 |  |
| 30.55 | Tetradecenal | |  | | | - |  | |  | | - | |  | |  | | | - |  | 0.04 | | + | 0.13 |  |
| 32.79 | Octadecenal? | |  | | | - |  | |  | | - | |  | |  | | | - |  | 0.04 | | + | 0.13 |  |
| 33.29 | Hexadecanal | |  | | | - |  | |  | | - | |  | | 0.12 | | | + | 0.17 | 0.82 | | + | 0.58 |  |
| 35.09 | Octadecanal | |  | | | - |  | |  | | - | |  | | 0.11 | | | + | 0.20 | 0.41 | | + | 0.52 |  |
| 36.87 | Eicosanal | |  | | | - |  | |  | | - | |  | |  | | | - |  | 0.29 | | + | 0.22 |  |
| Ketones | | | | | | | | | | | | | | |  | | |  |  |  | |  |  |  |
| 31.23 | Heptadecanone | | 0.05 | | | + | 0.08 | |  | | - | |  | |  | | | - |  | 0.45 | | + | 0.32 |  |
| 34.94 | Nonadecanone | | 0.04 | | | + | 0.06 | |  | | - | |  | |  | | | - |  |  | | - |  |  |
| Other | | | | | | | | | | | | |  | |  | | |  |  |  | |  |  |  |
| 35.21 | 2 (3h)-furanone. 5-dodecyldihydro- | | 0.03 | | | + | 0.04 | | 0.03 | | + | | 0.03 | |  | | | - |  |  | | - |  |  |
| 36.79 | 9-octadecenamide | |  | | | - |  | | 0.01 | | + | | 0.01 | |  | | | - |  |  | | - |  |  |
| 38.73 | Unidentified furanone | |  | | | - |  | | 0.00 | | + | | 0.00 | |  | | | - |  |  | | - |  |  |
| 39.67 | 13-docosenamide | | 0.08 | | | + | 0.09 | | 0.02 | | + | | 0.05 | |  | | | - |  |  | | - |  |  |
| 50.06 | α-Tocopherol | | 0.06 | | | + | 0.09 | | 0.07 | | + | | 0.04 | |  | | | - |  |  | | - |  |  |
