# Supplementary material for: Seasonal and interpopulational phenotypic variation in morphology and sexual signals of Podarcis liolepis lizards
Source: PLoS One. 2019 Mar 15;14(3):e0211686. doi: 10.1371/journal.pone.0211686 (PMC6419997; doi:10.1371/journal.pone.0211686)
Supplement: S7 Table — PL codes refer to P.liolepis individuals sampled in the Midi-Pyrenees. Med1 and Bur2 (from Medinaceli and Burgos, respectively) and MT1 (P. muralis) are sequences obtained from GenBank. (DOCX) [file pone.0211686.s007.docx]

| **Code** | | **GenBank accession number** | |
| --- | --- | --- | --- |
| PL3 | MK160635 | |  |
| PL4 | MK160636 | |  |
| PL5 | MK160637 | |  |
| PL10 | MK160638 | |  |
| PL11 | MK160639 | |  |
| PL12 | MK160640 | |  |
| PL14 | MK160641 | |  |
| PL16 | MK160642 | |  |
| PL13 | MK160643 | |  |
| PL15 | MK160644 | |  |
| PL6 | MK160645 | |  |
| PL7 | MK160646 | |  |
| PL8 | MK160647 | |  |
| PL51 | MK160648 | |  |
| PL53 | MK160649 | |  |
| PL21 | MK160650 | |  |
| PL23 | MK160651 | |  |
| PL24 | MK160652 | |  |
| PL25 | MK160653 | |  |
| PL26 | MK160654 | |  |
| PL27 | MK160655 | |  |
| PL28 | MK160656 | |  |
| PL49 | MK160657 | |  |
| PL50 | MK160658 | |  |
| PL54 | MK160659 | |  |
| PL57 | MK160662 | |  |
| PL58 | MK160663 | |  |
| PL59 | MK160664 | |  |
| PL60 | MK160665 | |  |
| PL61 | MK160666 | |  |
| PL70 | MK160667 | |  |
| Med1 | DQ081167 | |  |
| Bur2 | DQ081166 | |  |
| MTA | DQ081182 | |  |
